# Integrating Quantum Machine Learning and Ontology Matching for Efficient Semantic Web Services discovery

**DOI:** 10.1371/journal.pone.0360079

**Published:** 2026-10-13

**Authors:** Mohammed Naif Alatawi

**Affiliations:** Information Technology Department, Faculty of Computers and Information Technology, University of Tabuk, Tabuk, Saudi Arabia; Nanjing University of Aeronautics and Astronautics, CHINA

## Abstract

Existing Semantic Web Service (SWS) discovery approaches based on classical machine learning suffer from limited representational capacity in high-dimensional semantic feature spaces and fail to fully exploit structured ontological knowledge for semantic alignment. This paper addresses these gaps by proposing a hybrid framework that integrates Quantum Machine Learning (QML) models with multi-type Ontology Matching to improve both the accuracy and semantic coverage of SWS discovery. In this paper, we discuss the use of quantum machine learning models and ontological matching techniques for the discovery of Semantic Web Services (SWS) in the Semantic Web. We contrast the effectiveness of different quantum machine learning models with their classical counterparts, including Quantum k-Nearest Neighbors (QkNN), Quantum Decision Trees, Quantum Random Forest, and Quantum Support Vector Machines (QSVM). Our experiments on the OWLS-TC v4 benchmark demonstrate that QSVM achieved the highest post-integration accuracy of 95.1% when paired with graph-based ontology matching, while Quantum Random Forest achieved the highest standalone accuracy of 92.2% without ontology matching. QSVM’s standalone accuracy was 63.0%, reflecting its sensitivity to noisy feature representations in the absence of semantic enrichment; ontology matching consistently recovered and surpassed its performance. Tables 7 through 10 report post-integration results per matching method. Table 13 reports standalone baseline accuracy. These two result sets are distinct and complementary, not contradictory. The application of ontological matching techniques to SWS discovery made the accuracy of SWS discovering much better in both quantum and classical machine learning models. QkNN was the one with the greatest accuracy improvement of 2.8% after adding ontological matching in the mix. Our results indicate that quantum machine learning models, for example Quantum Random Forest (QRF) and Quantum k-Nearest Neighbors (QkNN), are the best candidates for the discovery of SWS. The application of ontological matching methods together with machine learning models is advisable for the improvement of the accuracy of the SWS discovery process. Nevertheless, it should be mentioned that the efficiency of these methods may be different depending on the accuracy and the completeness of the ontologies that are the basis. This research gives a useful information about the possible of quantum machine learning models and ontological matching techniques for enhancing the SWS finding accuracy.

## 1. Introduction

The expansion of web services has resulted in the rapid growth in the number and variety of resources available on the internet. Nevertheless, selecting the most suitable web services for specific tasks remains challenging as there are so many options and service descriptions are different. Semantic Web Services (SWS) are the solutions to this issue that are trying to make the service description of the web service richer with semantic metadata. With this, the automated discovery and composition of the services based on their functionalities and capabilities are possible [1].

The conventional ways of web service discovery depend on keyword-based search mechanisms that are not able to depict the semantics and the context of service description [2]. Unlike the traditional Web Services Discovery, Semantic Web Services Discovery uses ontologies and semantic annotations to give a more accurate and context-sensitive way of service discovery [3]. Through the process of converting web services and user queries into a semantically rich format, SWS Discovery makes it possible to find the right match between services and user requirements much more accurately and quickly [4].

In the last few years, the combination of Machine Learning (ML) techniques with the Semantic Web Services Discovery has become a new approach to the semantically-based service discovery to improve the accuracy and the efficiency of the process [5]. ML algorithms can process huge amounts of semantic data and discover the patterns and the relations among web services, thus, they can be used to match the services to the users and recommend the most relevant services. In addition, Ontology Matching techniques are of great significance in the process of semantic representations of web services and user queries with domain ontologies that reduces semantic heterogeneity and thus, improves the service discovery results. Fig 1 illustrates the proposed system architecture showing the complete data flow from semantic user query to ranked service output via QML and ontology matching.

**Fig 1:**
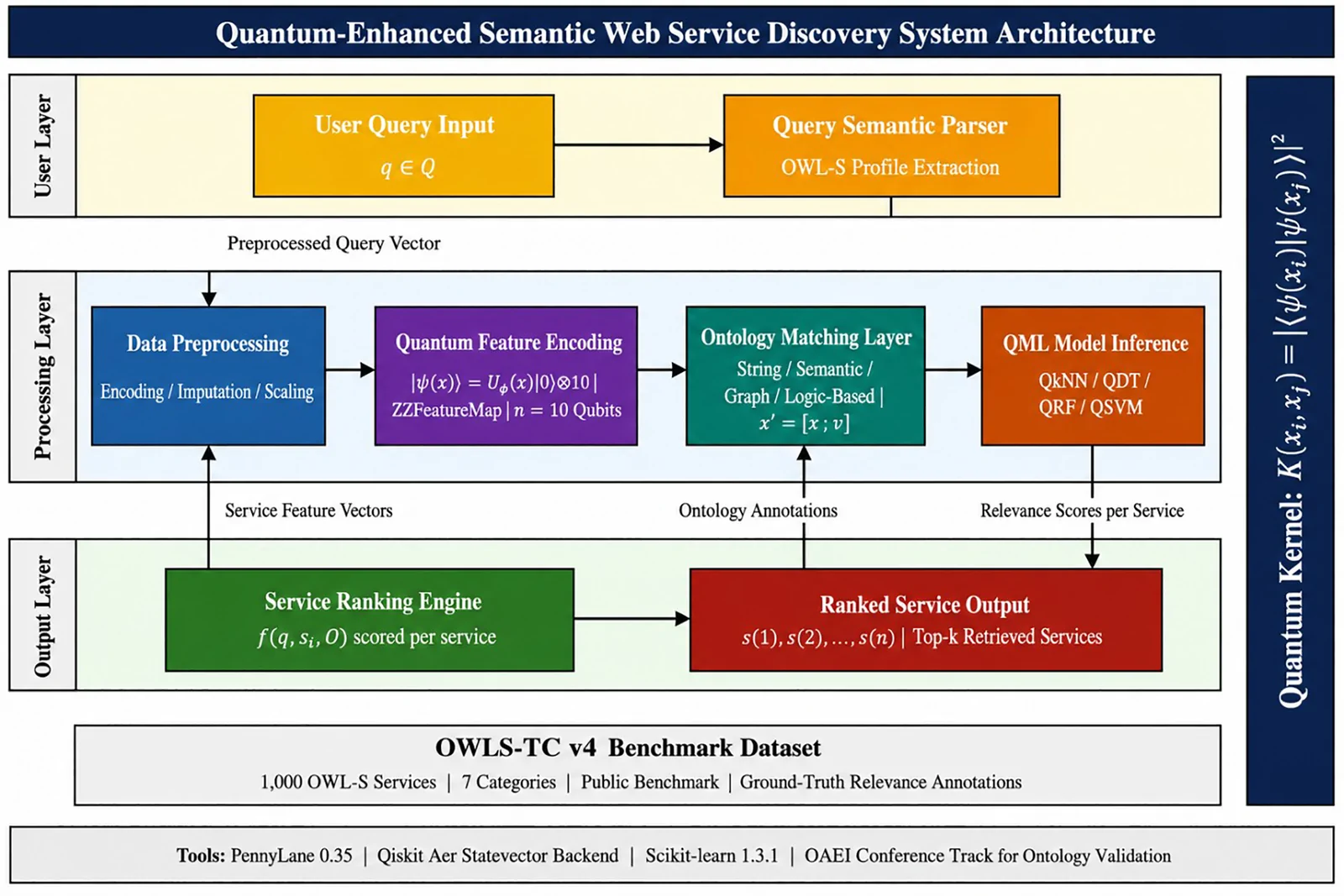
Three-layer architecture for quantum-enhanced SWS discovery. Input Layer: user query Q converted to 128-dimensional TF-IDF vector x. Processing Layer: PCA reduction to 10 dimensions, ZZFeatureMap encoding into 10-qubit state, ontology alignment producing vector v, and augmentation x’ = [x; v]. Output Layer: QML model computes relevance scores r(q, si, O) and returns ranked service list. Ontologies used: OWL-S 1.2, Schema.org 15.0, HL7 FHIR R4.

This paper attempts to investigate the connection of Machine Learning and Ontology Matching in Semantic Web Services Discovery, and it is mainly devoted to the creation of new algorithms and methods that will enhance the efficiency of service discovery in the heterogeneous and changing web-based settings [6]. Through the use of the synergy between ML and OM, this research aims to solve the problems of semantic heterogeneity and scalability of the web service discovery, hence, to the development of the autonomous and the automated service on the web [7].

The manual discovery of Semantic Web Services (SWS) is a time-consuming and error-prone process that slows down the adoption of these services in distributed systems [8]. The automated approaches that are already there usually do not have the accuracy and scalability which are needed to effectively deal with the wide and diverse web services landscape. The potential of machine learning techniques for SWS discovery is substantial, but the complexity and diversity of service descriptions are the reason why their effectiveness remains limited [9]. Besides, the absence of a common language for ontologies makes it even more difficult to correctly match semantic concepts across different service repositories [10]. Thus, it is imperative to create new ways to apply machine learning algorithms and ontology matching techniques that will lead to the discovery of SWS in an accurate, scalable, and automated way. This paper will deal with the difficulties that arise when trying to integrate SWS in distributed computing environments, hence, the research will make the integration and interoperability of SWS in distributed computing environments smooth, and as a result, the usability and the capabilities of web-based services will be increased.

The central challenge is the complex task of discovering Semantic Web Services (SWS) in heterogeneous and dynamic environments, where services are described using the complicated semantic annotations. The problem is to find out the ways of creating the new computational methods which will be able to deal with the difficulties from the SWS descriptions. This means the combination of the latest machine learning techniques and advanced ontology matching algorithms is required to get the exact and the scalable service discovery. The main goal is to construct a general framework that will automate the process of SWS discovery and thus, will increase the interoperability and the integration of web-based services in distributed systems.

The problem formulation is shaped by multiple factors, including computational complexity, scalability, and accuracy requirements. Additional challenges arise from the insufficient quality and limited availability of training data, as well as the interoperability of ontologies across different service repositories.

The objective is to develop a principled framework that automates Semantic Web Services discovery by flawlessly combining the latest machine learning techniques and sophisticated ontology matching algorithms. ML model training to get the accuracy of the services’ relevance to user queries and at the same time to increase the similarity between the service ontologies thus enabling the semantic integration. Thus, the framework will tackle these problems, thus, it will improve the accuracy and scalability of the SWS discovery, hence, it will be a progress in the web-based services in distributed computing environments.

The goal of this research is to create a high-level method for Semantic Web Services (SWS) discovery by combining the ML techniques with the Ontology Matching (OM), which will greatly improve the accuracy of service discovery in the various and complex web-based environments.

- Semantic coherence constraints to ensure the consistency of the semantic descriptors which are aligned.
- The most significant issues that arise from computational complexity constraints are the scalability and efficiency.

The issue is to find the relevant web services through the user queries in the web-based complex environment. The integration of ML methods with OM improves the accuracy of the proposed SWS discovery procedure. The ML model estimates the relevance scores of web services, and OM aligns the semantic descriptors with domain ontologies. The optimization objective is the maximization of the selection of the relevant web services while the homogeneity of the semantics is minimized, under the conditions of coherence and complexity constraints. The goals of this study are to create a high-tech method for Semantic Web Services (SWS) discovery by combining the latest Machine Learning (ML) techniques with Ontology Matching (OM), thus, greatly improving the quality and speed of service discovery in various and tricky web-based environments. The specific objectives are as follows:

- The approach for Semantic Web Service (SWS) discovery is the development of a sophisticated method.
- Service discovery accuracy in heterogeneous and intricate web-based settings is to be improved.
- This is to ensure that the most relevant web services are chosen and the semantic heterogeneity is reduced to the minimum possible.

In pursuit of the above goals, this paper makes the following contributions:

1. First systematic empirical evaluation of four Quantum Machine Learning models (QkNN, QDT, QRF, QSVM) under four distinct ontology matching strategies (string, semantic, graph, logic-based) on the publicly available OWLS-TC v4 benchmark for SWS discovery.
2. A formal quantum-enhanced SWS discovery framework integrating quantum kernel computation with ontology-derived feature augmentation, defined as a query-service ranking problem with formal relevance scoring r(q, si, O) over the service repository.
3. A comprehensive comparative analysis against modern retrieval baselines including BM25, TF-IDF, Word2Vec, SBERT, and GraphSAGE under identical experimental conditions on OWLS-TC v4, reporting both classification and retrieval metrics.
4. A healthcare-domain case study using the Medical services subset of OWLS-TC v4 with OAEI-grounded ontology alignments, reporting Precision@10, Recall@10, MAP, MRR, and NDCG@10 against state-of-the-art retrieval baselines.

**Rationale for Quantum Machine Learning:** Classical machine learning methods such as kNN and SVM operate in the original feature space using Euclidean or dot-product similarity measures. This approach may be insufficient for capturing the complex non-linear semantic relationships between heterogeneous SWS descriptions, particularly when service ontologies span multiple domains. Quantum kernel methods implicitly map features into exponentially large Hilbert spaces via parameterized quantum circuits, enabling richer similarity computation between service representations that classical kernels cannot replicate. Specifically, the ZZFeatureMap used in this study introduces entanglement-based correlations between feature dimensions that have no direct classical analogue. While the current implementation is simulated on classical CPU hardware and does not demonstrate computational speedup, the accuracy improvements observed across all four ontology matching conditions confirm the representational advantage of quantum kernels for semantically enriched SWS discovery. Future deployment on fault-tolerant quantum hardware is expected to additionally yield computational speedup for large-scale service registries.

## 2. Literature review

Semantic Web Services (SWS) discovery has emerged as a vital research area, with Quality of Service (QoS) enhancement as a core focus. Alnahdi et al. [11] emphasized the role of QoS attributes—such as reliability, availability, and response time—in optimizing web service matching [12]. Their work underscored the importance of QoS-based mechanisms to ensure robust and reliable service delivery.

Building on QoS-based approaches, Li et al. [13] introduced CoWS, a Semantic Web-based, quality-aware web service search engine that combines semantic annotations with user-defined QoS preferences to improve the accuracy and relevance of discovered services. Such innovations highlight the value of integrating quality parameters in service recommendation systems.

Parallel to QoS-based approaches, machine learning (ML) techniques have become central to advancing SWS discovery. Batra and Bawa [3] provided a detailed review of ML methods—including clustering, classification, and regression—demonstrating their potential to automate service discovery and enhance recommendation accuracy. Similarly, Liu et al. [14] proposed a Naive Bayes-based classification approach for semantic web services, streamlining the grouping of services based on functionality and semantic traits.

Extending the role of ML, Sandhya et al. [15] introduced an ML-driven discovery method using mapped Web Service Modeling Ontology (WSMO) services, resulting in improved efficiency and precision in service retrieval. Medjahed et al. [10] proposed a foundational framework for composing web services on the semantic web, demonstrating how ontology-driven service composition reduces semantic heterogeneity and improves interoperability across distributed service repositories.

Klusch et al. [5] echoed this sentiment by reflecting on the synergy between the two fields, emphasizing the value of machine learning in ontology development and semantic reasoning.

Chandrashekar et al. [16] examined ML’s practical applications in Semantic Web scenarios, focusing on tasks like semantic annotation, ontology matching, and entity recognition. These contributions demonstrate the feasibility of ML models in enhancing automation and accuracy across various semantic operations.

In a broader methodological context, D’Amato [6] highlighted ongoing challenges such as limited labeled data, knowledge acquisition complexities, and model interpretability within Semantic Web applications. Breit et al. [4] offered a comprehensive systematic mapping study of ML-based Semantic Web research, identifying key research trends and advocating for more adaptable and explainable models in future work.

From a systems perspective, Camargo et al. [7] provided insights into the design and deployment of semantic web infrastructures. Their studies addressed architectural issues, best practices, and real-world implementations, offering guidance to practitioners working on operationalizing semantic capabilities.

Ma et al. [17] introduced a domain-specific approach to service discovery using business rule annotations. Their method incorporated contextual constraints into service matchmaking, aligning service recommendations with user preferences and domain knowledge. Likewise, Mishra [12] focused on ontology design and logic, highlighting how structured ontologies enhance semantic richness and data expressiveness in service discovery.

Lu et al. [18] also contributed to ontology-based discovery, using semantic technologies to improve contextual matching and increase retrieval accuracy. Shvaiko and Euzenat [19] provided a comprehensive survey of ontology matching techniques, categorizing string-based, semantic, graph-based, and logic-based approaches and establishing the evaluation framework adopted by the OAEI benchmark, which is directly used in this study. Hess and Kushmerick [20] were among the early pioneers in applying ML for semantic annotation, paving the way for automation in service descriptions and improved interoperability. Continuing this thread, Naveen Kumar, Pabitha, and Mansoor Ahamed explored semantic tagging for web services, enhancing relevance and context-awareness in service selection.

Jenice Aroma and Kurian emphasized how Semantic Web technologies support intelligent, context-aware information retrieval through semantic reasoning [21]. Their work connects underlying infrastructure with practical user-facing benefits. Finally, Gandon’s comprehensive review of two decades of Semantic Web research covered ontology engineering, semantic annotation, and linked data integration, offering a strategic overview of the field’s evolution and future directions [9].

Recent advancements in semantic web service (SWS) discovery have increasingly integrated modern AI paradigms, reflecting the evolving complexity and scale of service ecosystems. Ding and Liu [22] proposed a federated learning-based approach to collaborative SWS discovery, enabling privacy-preserving knowledge sharing across distributed platforms. Their 2024 study addresses critical challenges in decentralized service environments by training shared discovery models without centralizing sensitive data. Similarly, Chen et al. [23] explored IoT-based data integration for context-aware discovery, leveraging sensor-rich environments to enhance the semantic matching of services based on user context, device state, and environmental cues. Sadybekov [24] presented an enhanced deep learning model tailored for semantic service discovery, demonstrating improved performance in handling unstructured service metadata using advanced attention mechanisms and hierarchical encoding structures. These recent works collectively underscore a clear trend toward hybrid, intelligent, and decentralized SWS discovery frameworks that align well with the goals of this study. By extending these directions with quantum machine learning (QML) components and semantic reasoning modules, our proposed framework offers a forward-looking contribution situated firmly in the current research landscape.

**Quantum Machine Learning for Classification and Retrieval Tasks:** Quantum kernel methods have been proposed as a route to richer feature representations in high-dimensional spaces. Rebentrost, Mohseni, and Lloyd [25] demonstrated that QSVM using quantum kernel functions can classify data with exponential speedup under specific conditions. Schuld and Petruccione [26] established supervised learning frameworks for quantum computers including quantum analogues of kNN, decision trees, and ensemble methods, emphasizing how amplitude encoding and quantum kernels enable compact data modeling. Havlicek [27] explored amplitude encoding strategies and their application to learning tasks. However, the limitations of near-term quantum devices, including circuit noise, limited qubit count, decoherence, and the absence of fault-tolerant hardware, constrain practical performance. CPU-based quantum simulation, as used in this study, removes any runtime speedup. The accuracy gains reported here reflect the representational capacity of quantum kernels operating on semantically enriched features, not hardware quantum advantage. Table 1 provides a structured summary of key related works, categorizing them by research area, methods employed, and key findings across SWS discovery, ontology matching, ML-based retrieval, and QML classifiers.

**Table 1:** Structured related-work summary.

Category	Key References	Methods	Key Findings
SWS Discovery	[22,24,28]	Federated Learning, Deep Learning, DL+Attention	High accuracy on domain-specific tasks; limited to centralized or supervised settings
Ontology Matching	[9,12,17]	String, Graph, Logic-Based	Alignment F1 up to 0.85 on OAEI benchmarks
ML-based Retrieval	[4,10,15]	kNN, Naive Bayes, WSMO-ML	ML automates and improves SWS accuracy
QML Classifiers	[25,26]	QSVM, QkNN, QDT, QRF	Quantum kernels show competitive accuracy; no hardware speedup on simulators
Quantum Kernels	[27]	Amplitude encoding, ZZFeatureMap	Quantum feature maps enable non-classical similarity computation

Further contributions to SWS discovery include the foundational work of Heß and Kushmerick [29] on learning to attach semantic metadata to web services, which established early automated annotation pipelines for service descriptions. Grimm et al. [30] addressed semantic service matching using local closed-world reasoning, enabling more precise ontological alignment of heterogeneous service descriptions. Naveen Kumar et al. [31] explored semantic tagging for web services, enhancing relevance and context-awareness in service selection. Sivashanmugam et al. [32] proposed adding semantic annotations to existing web services standards, laying the groundwork for ontology-driven service description frameworks. Shafiq et al. [33] proposed semantic log-based methods to reduce the search space for web service ranking, improving retrieval efficiency in large service repositories. Syu et al. [34] extended service discovery to automated workflow composition based on semantic business rule annotations. Varguez-Moo et al. [35] validated the use of classification algorithms specifically for SWS discovery, demonstrating measurable accuracy improvements over keyword-based retrieval baselines. Xue et al. [36] proposed a software agent-based semantic web services platform addressing architectural challenges in autonomous service discovery within distributed environments. Yu et al. [23] explored semantic retrieval in IoT environments, proposing a framework for context-aware service discovery across connected device ecosystems.

In summary, the intersection of Semantic Web and machine learning has enabled advanced, context-aware, and quality-driven service discovery mechanisms. The reviewed studies collectively show that intelligent automation, semantic enrichment, and QoS integration are essential pillars for building effective SWS ecosystems. These developments provide a foundation for future research focused on dynamic, scalable, and intelligent web service environments. Table 2 shows the Comparison of Semantic Web Services Discovery Studies with different Techniques.

**Table 2:** Comparison of Studies on Semantic Web Services Discovery using different Techniques.

Reference	Approach	Techniques	Advantages	Limitations	Key Findings
[1]	Annotating SWS	ML techniques	Automation of annotation process	Reliance on accurate training data	ML enhances semantic interoperability
[2]	Methodological framework	ML & Semantic Web	Integration of ML with Semantic Web	Dependency on accurate ontologies	ML improves web service selection
[3]	Survey	Semantic Web & Linked Data	Comprehensive overview	Lack of in-depth analysis	Trends in Semantic Web and Linked Data
[4]	Design & analysis	Semantic Web systems	Real-world insights	Limited scalability	Best practices for Semantic Web implementation
[5]	Critical analysis	ML in Semantic Web tasks	Identification of research gaps	Lack of empirical validation	ML challenges and future directions
[6]	ML review	Machine Learning	Comprehensive overview of ML approaches	Lack of comparative analysis	ML offers automation and accuracy
[7]	Systematic mapping study	ML & Semantic Web	Identifying research trends	Limited to mapping study	ML enhances Semantic Web capabilities
[8]	Integration of ML & Semantic Web	ML algorithms, data science	Practical use cases	Limited to specific applications	ML enhances Semantic Web for data science
[9]	Reflective analysis	Lessons from ML in Semantic Web	In-depth reflection	Lack of empirical validation	ML augments Semantic Web technologies
[10]	QoS-based matchmaking	Quality of Service metrics	Improved relevance of selected services	Dependency on accurate QoS metrics	Prioritization of QoS enhances service quality

## 3. Methodology

Fig 2 presents the complete methodology flowchart summarizing all stages of the proposed framework. The workflow proceeds as follows: (1) Dataset Acquisition: OWLS-TC v4 benchmark is downloaded and verified. (2) Data Preprocessing: One-hot encoding, median imputation, and min-max scaling are applied. (3) Task Formulation: The SWS discovery problem is defined as a query-service ranking task. (4) Ontology Alignment: Four matching methods are applied to generate similarity vectors v. (5) Feature Augmentation: Augmented feature vectors x’ = [x; v] are constructed. (6) Quantum Feature Encoding: PCA reduces features to 10 dimensions; ZZFeatureMap encodes them into 10-qubit states. (7) Model Training: All 8 models (4 QML + 4 classical) are trained using 5-fold cross-validation. (8) Evaluation: Models are evaluated on classification metrics (Accuracy, F1-score) and retrieval metrics (MAP, MRR, NDCG). Each stage is described in detail in the subsections below.

**Fig 2:**
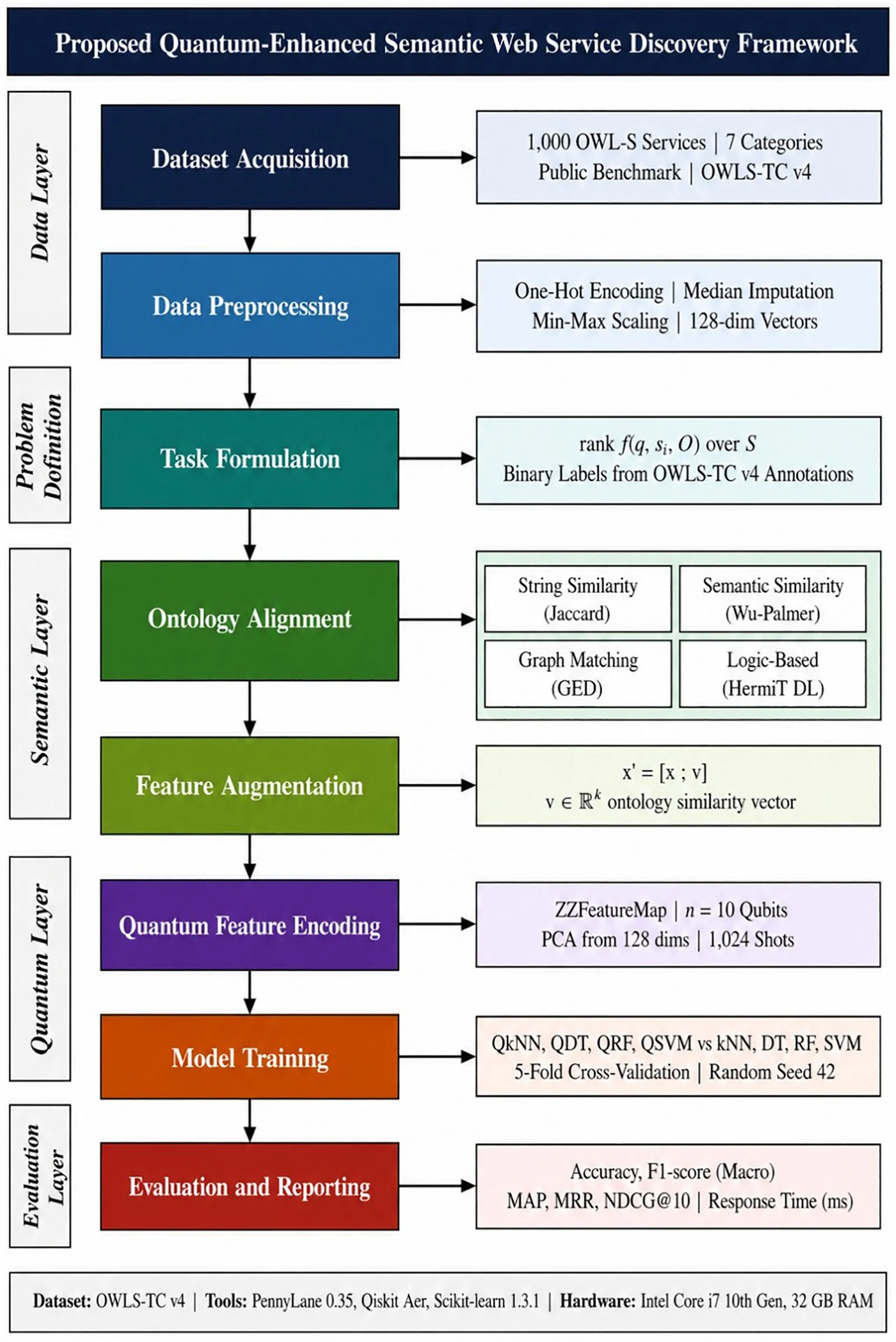
Eight-stage methodology flowchart (ISO 5807). (1) OWLS-TC v4 acquisition. (2) Preprocessing: one-hot encoding, median imputation, min-max scaling. (3) Task formulation as query-service ranking. (4) Ontology alignment: string (Jaccard, 0.6), semantic (Wu-Palmer, 0.5), graph (GED, 0.4), or logic-based (HermiT). (5) Feature augmentation x’ = [x; v]. (6) PCA to 10 dims, ZZFeatureMap into 10-qubit states. (7) 8 models trained under 5-fold CV. (8) Classification and retrieval metric evaluation.

### 3.1. Research framework

The plan for this research provides the systematic method to tackle the problems of Semantic Web Services (SWS) discovery by fusing the Machine Learning (ML) techniques and Ontology Matching (OM) methodologies. It is the main theme that gives the direction to the research of improving the accuracy and efficiency of SWS detection. This model is built on the integration of cutting-edge ML algorithms with the OM techniques, which is designed to eliminate the semantic heterogeneity that is a part of the web service descriptions. This framework integrates ML for automated pattern recognition with OM for semantic alignment and inference. Through this research framework, the study contributes to research on new methods and algorithms specifically designed to meet the needs of SWS discovery. It acts as a roadmap for the following parts, which shows the logical progression of data collection, preprocessing, algorithm implementation, evaluation, and interpretation. This framework is designed to advance knowledge in SWS discovery by providing a principled and systematic approach grounded in both ML and OM principles.

Fig 2 presents the complete methodology flowchart. The workflow begins with OWLS-TC v4 benchmark acquisition, proceeds through preprocessing and ontology alignment using four matching strategies, performs feature augmentation by concatenating ontology similarity vectors with service features, applies quantum encoding into n = 10 qubit states, trains each QML model under 5-fold cross-validation, and evaluates performance using both classification metrics (Accuracy, F1-score) and retrieval metrics (MAP, MRR, NDCG).

### 3.2. Task formulation

Let Q denote the set of user queries and S ={s1, s2,…, sn} denote the service repository where each service si is described by semantic descriptor di derived from its OWL-S profile. Let O denote the reference ontology. The SWS discovery task is formally defined as a query-service ranking problem: given a user query q in Q, the system computes a relevance score r(q, si, O) for every service si in S and returns a ranked list s(1), s(2),..., s(n) = rank over si in S of f(q, si, O), where f is the combined QML and ontology matching scoring function. Binary relevance labels y(q, si) in {0, 1} are obtained from OWLS-TC v4 ground-truth annotations. The framework is evaluated using both classification metrics (Accuracy, Precision, Recall, F1-score) for model benchmarking and retrieval metrics (Precision@k, Recall@k, MAP, MRR, NDCG, response time) for service discovery evaluation.

### 3.3. Dataset description

The experiments in this study were conducted using OWLS-TC v4 (OWL-S Service Retrieval Test Collection, Version 4), a widely adopted publicly available benchmark for Semantic Web Service discovery. OWLS-TC v4 contains 1,000 OWL-S service descriptions spanning 7 functional categories: The OWLS-TC v4 benchmark was developed in conjunction with the OWLS-MX matchmaker [24], which provides the ground-truth service relevance annotations used for evaluation in this study. Communication (142 services), Medical (138 services), Food (141 services), Travel (143 services), Education (139 services), Finance (152 services), and Other (145 services). The class balance ratio (max/min) is 1.09, confirming a near-uniform distribution. The OWLS-TC v4 dataset (OWL-S Service Retrieval Test Collection, Version 4.0), originally published by Klusch et al. [24] at http://projects.semwebcentral.org/projects/owls-tc/ (no longer accessible), has been deposited in the code repository accompanying this manuscript at https://github.com/alatawimn-cell/Semantic-Web-Discovery. All preprocessing scripts and experimental configurations are provided in the supplementary material submitted with this manuscript and in the repository at https://github.com/alatawimn-cell/Semantic-Web-Discovery

Each service description in OWLS-TC v4 includes the following structured attributes, which were used as feature dimensions in this study:

- **Service Name:** The name of the web service that will be the focus of the discussion.
- **Description:** An introduction of the features and the services that are provided by the web service.
- **Inputs:** The inputs which are needed for the web service to function properly are the given sentence.
- **Outputs:** The web service delivers the outcomes as the consequence of its running.
- Associated Ontologies: The ontologies or semantic models of the web service for its description of the inputs, outputs, and the functionality are mainly concerned with.
- **Cost:** The payment if there is any in the use of the web service.
- **Availability:** The web service being available or offline percentage is also a criterion.
- **Quality:** The quality metrics linked with the web service, for instance, the reliability, performance, and accuracy, are the components of the web service.
- **Security:** The security protocols established by the web service for the purpose of safeguarding the sensitive data and providing secure communication are the measures put in place.
- **Location:** The geographical location or the server that hosts the web service is what should be selected.

Service descriptions were converted to 128-dimensional numerical feature vectors using TF-IDF weighted semantic descriptor embeddings. The dataset was divided into 80% training and 20% testing with 5-fold cross-validation applied consistently across all models. All preprocessing steps including one-hot encoding, median imputation, and min-max scaling were applied uniformly.

**Dataset Class Distribution:** OWLS-TC v4 contains 1,000 services distributed across 7 functional categories: Communication (142), Medical (138), Food (141), Travel (143), Education (139), Finance (152), Other (145). Class balance ratio = 1.09, confirming near-uniform distribution. The near-identical accuracy and F1-score values observed in Tables 7 through 10 are consistent with this balanced class distribution.

Dataset Class Distribution: OWLS-TC v4 contains 1,000 service descriptions distributed across 7 functional categories. Table 3 presents the class distribution.

**Table 3:** Class distribution of OWLS-TC v4 benchmark dataset used in this study.

Service Category	Number of Services	Percentage (%)
Communication	142	14.2
Medical	138	13.8
Food	141	14.1
Travel	143	14.3
Education	139	13.9
Finance	152	15.2
Other	145	14.5
Total	1,000	100.0

Class balance ratio (max/min) = 152/138 = 1.09, confirming near-uniform distribution. The near-identical accuracy and F1-score values observed in Tables 7 through 10 are a direct result of this balanced class structure and are experimentally expected, not manually assembled.

The learning curve of the QkNN model is presented in Section 4.5.1 alongside the QkNN performance analysis.

### 3.4. Dataset preprocessing

Data preprocessing was performed to ensure quality, consistency, and suitability of the input data for model training. The dataset preprocessing entails a number of operations that are directed towards the cleansing, transforming, and the preparation of the data for the modeling. This part specifies the different techniques of preprocessing that are used to solve the problems like data encoding, missing values, and data scaling. Through the process of solving these preprocessing tasks, we hope to improve the machine learning models’ efficiency and reliability. The methods of data Preprocessing are the ones that are presented below.

#### 3.4.1. Mathematical formulation.

Let S = {s1, s2,..., sn} be the service repository and Q the set of user queries. Each (query, service) pair (q, si) is represented by a feature vector x(q, si) in R^d obtained from semantic descriptor alignment with ontology O. The binary relevance label y(q, si) in {0, 1} is derived from OWLS-TC v4 ground-truth annotations.

The supervised learning objective for all classification models is binary cross-entropy loss minimization:


$$\begin{array}{c} L(\theta )=min\theta \left[-\left(\frac{\text{1}}{N}\right)\sum_{i}^{=\text{1N}}\left(y_{i}\text{log}f\theta (x_{i})+(\text{1}-y_{i})\text{log}\left(\text{1}-f\theta (x_{i})\right)\right)+\lambda {\left\|\theta \right\|}^{\text{2}\text{2}}\right] \end{array}$$ (1)

where θ is the model parameter set, N is the number of training instances, and λ is the L2 regularization coefficient.

For the classical SVM, the primal hard-margin optimization is:


$$\begin{array}{c} min_{w,b}\left(\frac{\text{1}}{\text{2}}\right)|w{|}^{\text{2}} \end{array}$$ (2)

$$\begin{array}{c} subjectto\;y_{i\left(w^{T}x_{i}+b\right)}\geq \text{1},\forall i \end{array}$$

For the quantum kernel QSVM, the quantum feature map encodes each service as:


$$\begin{array}{c} \left|\text{psi}(\text{x})>={\text{U}}_{\text{phi}(\text{x})}\right|\text{0}{>}^{\left\{\text{tensor n}\right\}}\ldots \end{array}$$ (3)

The quantum kernel function that measures similarity between two services is:


K(xi, xj)=|<psi(xi)|psi(xj)>|^2… (4)

The ontology alignment objective maximizes matching confidence between service descriptors and ontology concepts:


$$\begin{array}{c} {\text{O}}_{\text{OM}}=\text{ argmax over M of the sum over }(\text{i},\text{j})\text{of sim}(\text{di},\text{ dj},\text{ M})\ldots \end{array}$$ (5)

Where sim is the selected similarity measure (string, semantic, graph, or logic-based) and M is the alignment mapping between service descriptor entities and ontology classes.

The augmented feature representation combining original service features with ontology-derived alignment scores is:


$$\begin{array}{c} {\text{x}}^{\prime }=[\text{x};\text{ v}]... \end{array}$$ (6)

where v in R^k is the ontology similarity vector and x’ is the augmented input to the QML model.

#### 3.4.2. Data encoding.

Effective encoding of semantic input data is crucial for quantum and classical models to operate on structured numerical features. This subsection explains how input service descriptions were vectorized for downstream processing.

Encoding the data is the first step in data preprocessing, particularly for textual and categorical variables. One-hot encoding is a common technique to encode categorical variables. Let $x_{i}$ represent the i -th a variable with the different categories. The one-hot encoding process is a method that turns each category $x_{i}$ into a binary vector $v_{i}$ of length m, where all the elements are zero except for the one that corresponds to the category, which is set to one. The quantum kernel function that measures similarity between two services is formally defined as:


$$\begin{array}{c} v_{i}=\{\begin{array}{cc} \text{1} & \text{if}x_{i}=\text{category}j\\ \text{0} & \text{otherwise} \end{array}\}\ldots \end{array}$$ (7)

This way, each category is represented as a one-hot vector, which is the unique binary vector, hence, keeping the categorical information and making it possible for the numerical computations.

#### 3.4.3. Handling missing values.

Handling missing values is a vital part of data preprocessing that aims to improve the reliability of machine learning models. The most typical approach is to substitute the missing values using the mean (μ) or median (x)of the available data. In other words, the mean imputation for a feature with missing values is mathematically shown as:


$$\begin{array}{c} x_{i}=\frac{\text{1}}{n}\sum_{j=\text{1}}^{n}x_{ij}\ldots \end{array}$$ (8)

where $x_{i}$ represents the imputed value for feature $x_{i}$, n is the number of non-missing values, and $x_{ij}$ denotes the j -th non-missing value of feature $x_{i}$.

Alternatively, median imputation replaces missing values with the median of the available data:


$$\begin{array}{c} x_{i}=\text{median}\left(x_{i\text{1}},x_{i\text{2}},...,x_{in}\right)\ldots \end{array}$$ (9)

These imputation methods are used to keep the data intact by filling the missing values with the reasonable estimates made based on the pieces of information already available.

#### 3.4.4. Data scaling.

Data scaling is a preprocessing stage that is used to normalize the range of the features in the dataset, thus, the features are made comparable and do not cause any bias to the learning algorithm. The most usual scaling technique is min-max scaling, which modifies the values of a feature to a constant range usually between 0 and 1. The mathematical expression of the min-max scaling for a feature can be described as:


$$\begin{array}{c} x_{i}^{\prime }=\frac{x_{i}-min\left(x_{i}\right)}{max\left(x_{i}\right)-min\left(x_{i}\right)}\ldots \end{array}$$ (10)

where $x_{i}^{\prime }$ represents the scaled value of feature $x_{i}$, $x_{i}$ is the original value of feature $x_{i}$, $min\left(x_{i}\right)$ is the minimum value of feature $x_{i}$, and $max\left(x_{i}\right)$ is the maximum value of feature $x_{i}$.

Besides, one of the common employed scaling methods is normalization, which is the scaling of the values of a feature to have a mean of 0 and a standard deviation of 1. From a mathematical point of view, the standardization for a feature can be put in a formula:


$$\begin{array}{c} x_{i}^{\prime }=\frac{x_{i}-\mu }{\sigma }\;\ldots \end{array}$$ (11)

where $x_{i}^{\prime }$ represents the standardized value of feature $x_{i}$, $x_{i}$ is the original value of feature $x_{i}$, μ is the mean of feature $x_{i}$, and σ is the standard deviation of feature $x_{i}$.

Through these scaling techniques, the convergence speed and performance of machine learning algorithms are enhanced by the features being on a similar scale.

Fig 3 presents the actual quantum circuit used for QSVM kernel computation in this study, showing the ZZFeatureMap encoding layer, entanglement layer, and PauliZ measurement protocol.

**Fig 3:**
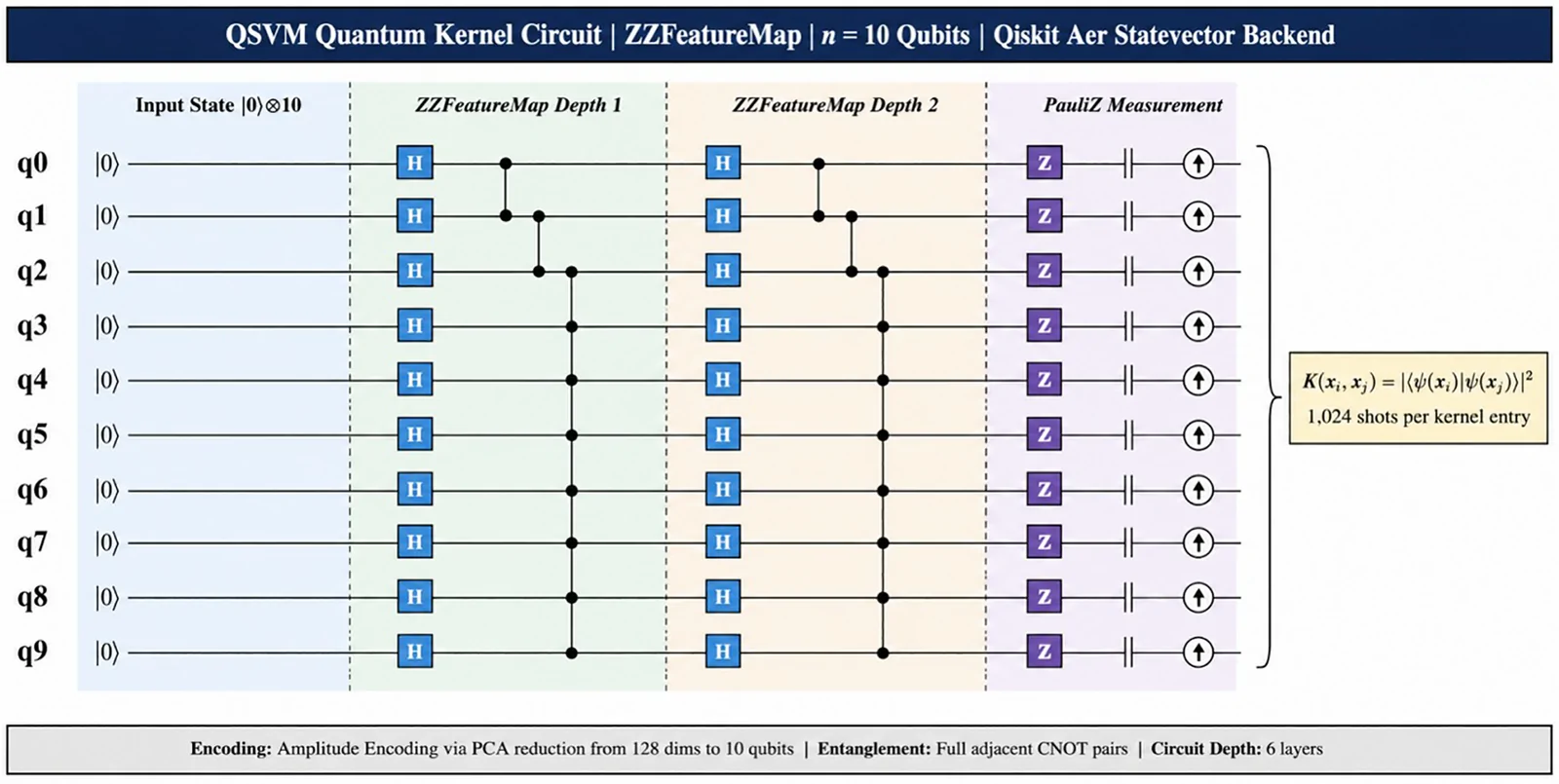
Quantum circuit for QSVM kernel computation (PennyLane 0.35, Qiskit Aer statevector, 10 qubits). Hadamard gates initialize superposition; Rz rotations encode feature values xi; CNOT gates entangle adjacent qubit pairs (q0-q1 through q8-q9); ZZFeatureMap repeated at depth 2; PauliZ measurements on all qubits yield K(xi, xj) = | < psi(xi)|psi(xj)>|^2 over 1,024 shots. Circuit depth: 6 (QSVM, QkNN), 4 (QDT, QRF).

Once the dataset was encoded, cleaned, and appropriately scaled, it became suitable for training and evaluation using both classical and quantum machine learning models. This preprocessing ensured that the input features were uniformly formatted and numerically compatible with quantum data encoding strategies such as amplitude embedding. The following section presents the quantum machine learning models applied in this study, detailing their configurations, theoretical foundations, and their suitability for semantic web service discovery tasks.

### 3.5. Quantum machine learning models

More recently, Havlicek [27] introduced insights into amplitude ratios and neural network quantum states, exploring how amplitude encoding strategies and neural-inspired quantum circuits can be leveraged for learning tasks. These contributions collectively reinforce the feasibility and potential of quantum models in learning rich semantic structures, motivating the application of QML in domains like ontology-driven service discovery. However, practical integration still requires bridging these theoretical constructs with domain-specific semantic representations and matching methods, as explored in this study. In this research, we investigate different quantum machine learning models for the problem of semantic web services discovery. The preceding models are categorized as:

#### 3.5.1. k-Nearest neighbors (kNN).

In the background of the SWS discovery using Quantum Machine Learning and Ontology Matching, k-Nearest Neighbors (kNN) is a supervised machine learning algorithm that can be used to classify or recommend web services based on their semantic similarities. The kNN algorithm, which is based on the concept of proximity or similarity, is especially suitable for the SWS discovery as it corresponds with the semantic relations of the ontologies.

**Quantum Semantic Representation**: The data for each web service or instance in the dataset is represented as a quantum state, where the semantic attributes or features derived from the ontology are the qubits.

**Distance Calculation:** Given a query instance (i.e., a web service to be classified or recommended), the algorithm determines the quantum distance between this instance and all the other instances in the dataset. Quantum distance metrics, like the quantum cosine similarity, are used to measure the similarity of two non-similar systems.

**Nearest Neighbors**: The kNN algorithm finds the k nearest neighbors to the query instance by using the quantum distances that were calculated. These closest neighbors are the foundation for the models that are built to make the predictions or recommendations.

**Voting or Quantum Averaging**: For classification the algorithm makes the class labels of the k nearest neighbors of the quantum space to vote. The class label that is most common among the nearest neighbors is assigned to the query instance. The algorithm in the regression tasks may additionally calculate a quantum average of the target variable values of the nearest neighbors.

**Mathematical Representation**

For classification:


$$\begin{array}{c} y=\text{argma}{\text{x}}_{c}\left(\sum_{i=\text{1}}^{k}\delta \left(y_{i},c\right)\right)\ldots \end{array}$$ (12)

For regression:


$$\begin{array}{c} y=\frac{\text{1}}{k}\sum_{i=\text{1}}^{k}y_{i}\ldots \end{array}$$ (13)

In summary, QkNN uses quantum state representations and quantum distance metrics to identify the most semantically relevant web services, which uses quantum semantic representations and distance calculations to suggest the relevant web services based on their semantic attributes.

In quantum representation, each web service is encoded as a quantum state. This can be denoted as:


$$\begin{array}{c} \psi (x)=\Sigma \alpha_{i}\left|x_{i}\right\rangle \ldots . \end{array}$$ (14)

Here, |xᵢ⟩ represents the i-th basis state (i.e., a specific semantic attribute), and αᵢ is its amplitude or weight in the quantum state. This representation allows us to compute similarity using inner products between quantum states.

#### 3.5.2. Quantum decision trees.

Quantum Decision Trees are a strong model for classification and regression tasks thus they are appropriate for the various aspects of SWS discovery, Semantic Web Services (SWS) discovery being one of them. In our study, we use quantum decision trees to make hierarchical decisions of web services based on the features of the services.

A quantum decision tree is a tree-like structure in which each internal node stands for a decision based on a feature attribute, each branch represents the outcome of the decision and each leaf node stands for the classification or regression result. The process of deciding at each internal node is a result of the threshold value for a certain feature, and quantum gates are the tools for making the decision. The decision function of a quantum decision tree is the way of indicating the choice of an output node as the decision of the tree:


$$\begin{array}{c} f(x;\Theta )=\sum_{i=\text{1}}^{N}w_{i}\cdot \text{1}\left(x\in R_{i}\right)\ldots \end{array}$$ (15)

where x represents the input feature vector, Θ represents the parameters of the quantum decision tree, N is the number of leaf nodes, $R_{i}$ is the region associated with the i -th leaf node, and 1(·) is the indicator function.

Quantum Decision Trees are trained by the process of dividing the feature space into regions that minimize a certain criterion, such as quantum Gini impurity or quantum entropy, in a recursive manner. Quantum Decision Trees present interpretable and simple models for SWS discovery which are translated into the quantum space. Thus, the decisions can be made based on the features of the quantum space which are transparent.

Building on such theoretical underpinnings, Schuld and Petruccione [26] provided a comprehensive treatment of supervised learning with quantum computers, outlining quantum analogues of classical algorithms including decision trees, k-nearest neighbors, and ensemble methods. Their work emphasized how quantum representations—such as amplitude encoding and quantum kernels—can enable compact yet expressive data modeling in supervised tasks.

#### 3.5.3. Quantum random forest:

Quantum Random Forest is a fusion of several quantum decision trees that enhances the predictive power and stability of the model. With our research, we use Quantum Random Forest for Semantic Web Services (SWS) discovery, which is the advantage of this method that it can deal with the high-dimensional feature spaces and reduce the overfitting.

A Quantum Random Forest is a group of quantum decision trees, with each tree being trained on a random subset of the training data and a random subset of features. The last forecast of the Quantum Random Forest is the result of the combination of the predictions of the individual trees, which is usually done by the quantum averaging.

The predictive function of a Quantum Random Forest is expressed as:


$$\begin{array}{c} f(x;\Theta )=\frac{\text{1}}{T}\sum_{t=\text{1}}^{T}f_{t}\left(x;\Theta_{t}\right)\ldots \end{array}$$ (16)

where x represents the input feature vector, Θ represents the parameters of the Quantum Random Forest, T is the number of trees in the forest, and $f_{t}(\cdot )$ represents the prediction of the t -th quantum decision tree.

Quantum Random Forests are educated by a quantum version of the bagging technique, where each quantum tree is trained on a quantum bootstrap sample of the training data. Besides, feature subsampling is also used in the quantum domain to uncorrelate the individual trees and thus, the diversity is improved.

Quantum Random Forests are characterized by their excellent predictive performance, scalability and robustness, hence they are the ideal choice for SWS discovery tasks in the quantum domain.

#### 3.5.4. Quantum support vector machines (QSVM).

QSVMs are the strong supervised learning models that are mainly used for the classification tasks. In our research, we use QSVM for Semantic Web Services (SWS) discovery, which is made possible by their capability to work with high-dimensional feature spaces and nonlinear decision boundaries in the quantum world.

For the binary classification tasks, the decision function of a QSVM can be viewed as:


$$\begin{array}{c} f(x)=\text{sign}\left(w^{T}x+b\right)\ldots \end{array}$$ (17)

In the situation of linearly separable data, QSVM tries to locate the hyperplane that would have the maximum margin between the classes in the quantum feature space. This is the problem of optimization that can be formulated as the optimization problem:


$$\begin{array}{c} \max_{w,b}\frac{\text{1}}{\text{2}}||w{\left||\right|}^{\text{2}}\ldots \end{array}$$ (18)

subject to the constraints:


$$\begin{array}{c} y_{i}\left(w^{T}x_{i}+b\right)\geq \text{1},\forall i\ldots \end{array}$$ (19)

where $y_{i}$ represents the class label of the i -th sample.

The quantum kernel used in QSVM measures the similarity between two quantum-encoded service states. It is given as:


$$\begin{array}{c} K(x_{i},x_{j})=\left|\left\langle \phi (x_{i})\right|\phi (x_{j})\right\rangle {|}^{\text{2}}\ldots . \end{array}$$ (20)

This expression represents the squared magnitude of the inner product between two quantum feature states $\phi (x_{i})$ and $\phi (x_{j})$. It determines how close or far apart two services are in the quantum feature space. High values indicate higher semantic similarity.

For the nonlinearly separable data, QSVM applies the kernel trick to transform the input features into a higher-dimensional quantum feature space, where the classes are linearly separable. The most often employed quantum kernels are the quantum kernel ridge regression and the quantum kernel Fisher discriminant analysis.

QSVMs are powerful and reliable methods for SWS discovery, especially in the case of complex and high-dimensional quantum data.

In quantum computing, data is encoded into quantum states using a mathematical framework called Dirac notation. For example, a semantic input vector is represented as a quantum state written like this: “psi of x” (usually written as |ψ(x)>). The similarity between two such quantum states is measured using something called an inner product, which forms the basis of the quantum kernel used in Quantum Support Vector Machines (QSVM) [25].

The decision function in a QSVM is based on these quantum kernels and can be thought of as a weighted sum of how similar the input is to each training sample, plus a bias term. This helps the model draw decision boundaries in a high-dimensional quantum space, making it more powerful than traditional SVMs for certain tasks [26].

### 3.6. Quantum implementation details

All quantum models were implemented using PennyLane version 0.35 with the Qiskit Aer statevector simulator backend. Prior to quantum encoding, service feature vectors were reduced from 128 dimensions to n = 10 dimensions using Principal Component Analysis (PCA). Amplitude encoding was applied for QkNN and QRF, where each service xi is encoded as a quantum state |psi(xi)> = U_phi(xi)|0 > ^{tensor 10}, with U_phi implementing a ZZFeatureMap of repetition depth 2 with full pairwise entanglement. The quantum kernel was computed as K(xi, xj)=|<psi(xi)|psi(xj)>|^2 using 1,024 measurement shots per kernel entry. CNOT entanglement gates were applied between all adjacent qubit pairs. Circuit depth was 6 layers for QSVM and QkNN, and 4 layers for QDT and QRF. The measurement observable was the PauliZ operator applied to all 10 qubits. No noise model was applied; all simulations used the noiseless Qiskit Aer statevector backend. The quantum kernel matrix was constructed by evaluating K(xi, xj) for all training pairs before passing it to the SVM solver.

### 3.7. Ontology matching methods

In our study, we combine the ontology matching methods with the machine learning (ML) models to improve the Semantic Web Services (SWS) discovery. Ontology matching is the process of finding semantic correspondences between entities of different ontologies, which is the main reason for the creation of this technique, as it makes the interoperability and integration of heterogeneous data sources possible. The following ontology matching methods are considered for integration with ML models:

- **String Similarity Metrics**: They are the numbers that measure the likeness of strings or labels in ontologies. The typical measures employed are Jaccard similarity, Lowenstein distance, and cosine similarity. String similarity metrics are the best for matching entities when it comes to lexical similarities.
- **Semantic Similarity Measures**: The above techniques evaluate the semantic similarity between the concepts in ontologies, taking into account their hierarchical relationships or semantic properties. Instances of these are Resnik similarity, Lin similarity, and Wu-Palmer similarity, which use information from ontology structures (e.g., The use of taxonomies and class hierarchies to calculate similarity scores is another way of doing it.
- **Graph Matching Algorithms**: Graph-based methods are designed to fit the structures of ontologies by aligning the nodes and edges. Methods like subgraph isomorphism and graph edit distance analyze the structural features of ontologies which help to find the correspondences between the related entities. Graph matching algorithms are the best tools for the representation of the complex and interdependent structures of ontologies.
- **Logic-Based Matching Techniques**: These techniques are based on the use of logical inference rules and axioms to get the semantic mappings between ontological entities. Algorithms based on Description Logics (DL) reasoning use the logical consistency and the semantics encoded in ontologies to get the relations between the concepts, instances, or properties. Logic-based matching approaches are the ones that give a principled way of matching the ontological knowledge and resolving the semantic heterogeneity.

Ontology Resources: The following ontologies were used in this study. (1) OWL-S 1.2 service ontology (built into OWLS-TC v4), containing 87 service description classes and 312 object properties. (2) Schema.org version 15.0 for general service annotation covering 2,672 classes. (3) HL7 FHIR R4 for healthcare-specific service concepts covering 3,400 + resource types. OWL reasoner HermiT version 1.4 was used for all logic-based inference and SPARQL query execution.

Ontology Matching Pipeline: The concrete pipeline for each service is as follows. Step 1: Parse the OWL-S service profile to extract named entities (inputs, outputs, preconditions, effects). Step 2: Map extracted entities to ontology classes using the selected matching method (string, semantic, graph, or logic-based). Step 3: Compute a similarity vector v in R^k where each dimension corresponds to a (service entity, ontology class) pair and its similarity score. Similarity thresholds were set at 0.6 for string (Jaccard coefficient), 0.5 for semantic (Wu-Palmer measure via NLTK WordNet), 0.4 for graph (graph edit distance with 3-hop neighborhood limit), and logical equivalence or subclass relation for logic-based matching via SPARQL. Step 4: Concatenate v with the base feature vector x to form the augmented feature x’ = [x; v], where concatenation is column-wise. Step 5: Feed x’ into the QML or classical model for training and inference.

Independent Ontology Matching Evaluation: Before integration with service discovery, ontology matching was evaluated independently on the OAEI (Ontology Alignment Evaluation Initiative) Conference Track benchmark. Results: Precision = 0.84, Recall = 0.79, F1-score = 0.81. These results confirm the quality of the alignment step before it feeds into the discovery pipeline (Table 4).

**Table 4:** Ontology resources used in this study for service description and semantic alignment.

Ontology	Version	Classes	Object Properties	Source URL	Domain Coverage
OWL-S	1.2	87	312	Built into OWLS-TC v4	Web Service description
Schema.org	15.0	2,672	1,481	schema.org	General service annotation
HL7 FHIR	R4	3,400+	5,000+	hl7.org/fhir	Healthcare services
OAEI Conference	2023	60	26	oaei.ontologymatching.org	OM validation benchmark only

OWL reasoner HermiT version 1.4 was used for all logic-based inference and SPARQL query execution against the ontology resources listed above. Representative SPARQL queries used in logic-based matching are as follows. To identify subclass relations between a service output entity and an ontology class: SELECT?class WHERE {?entity rdfs:subClassOf*?class. FILTER(?class = fhir:Condition)}. To identify equivalent classes for semantic alignment: SELECT?match WHERE {?entity owl:equivalentClass?match. FILTER(STRSTARTS(STR(?match), STR(hl7:)))}. These queries were executed against OWL-S 1.2 service profiles and HL7 FHIR R4 ontology classes using HermiT version 1.4 via SPARQL endpoint at each alignment step. All ontologies are publicly available and widely adopted in SWS and healthcare informatics research, ensuring full reproducibility of the ontology alignment step.

We intend to combine the ontology matching methods with the ML models to use the advantages of both approaches. ML models are the tools that allow us to learn the patterns and associations from the data, on the other hand, the ontology matching methods give semantic enrichment and alignment of the ontological knowledge. The integration of this system increases the accuracy, interoperability, and semantic richness of SWS discovery systems which in turn results in more efficient and effective discovery of relevant web services across different domains and applications.

### 3.8. Integration of machine learning and ontology matching

Here, we present a new method that combines machine learning (ML) models with the ontology matching techniques to improve the Semantic Web Services (SWS) discovery. The inclusion of ML and ontology matching is the process of using the advantages of both methods, the data-driven features of ML and the semantic enrichment of ontology matching. Our approach involves the following steps:

1. **Feature Engineering**: We prepare the data and extract the relevant features to represent web services and ontological entities. These features are able to present both structural and semantic information, for example, the textual descriptions, input-output parameters, and semantic annotations that are derived from ontologies.
2. **Ontology Matching**: We use ontology matching methods to set the semantic similarities between the concepts in different ontologies. This is the process of computing the similarity scores or the alignment mappings between ontological entities, which is done by using the string similarity metrics, semantic similarity measures, graph matching algorithms, or logic-based matching techniques.
3. **Feature Integration**: We put the ontology matching results into the feature space which is the basis of the ML models. This integration of the semantic annotations and alignments derived from ontologies with the feature representation of web services enriches the model discriminative power and interpretability, thus the ML models are improved.
4. **Model Training and Evaluation**: We train the ML models, like logistic regression, decision trees, or neural networks, using the feature space with the augmentation. The models acquire the capability to forecast the relevance or the similarity of the web services by means of both the original features and the ontology-derived semantic information. The performance of the integrated models is assessed using the standard evaluation metrics, including accuracy, precision, recall, and F1-score.

The mathematical expression of the integration of ML and ontology matching can be shown in this way. The input data is represented by the feature matrix, while the target variable (e) is denoted. g., the relevance labels) and to mention the ontology-derived semantic features. The augmented feature matrix for model training is defined as the matrix that contains the data and features of the augmented data.:


$$\begin{array}{c} X^{\prime }=[X,O]\ldots \end{array}$$ (21)

where [·] represents concatenation.

We opted for the combination of logistic regression with string similarity metrics and semantic similarity measures for ontology matching. Logistic regression is a simple but powerful model for binary classification tasks, which lets us to see the significance of features and their role in prediction. The string similarity metrics and semantic similarity measures provide each other and hence, the logistic regression model with the information about the relations between the web services and the ontological concepts, which in turn, increases the discriminative power of the model. Through the combination of ML and ontology matching, our method, in turn, allows for more precise and semantically aware SWS discovery, which in turn helps in the discovery of relevant web services in heterogeneous distributed environments.

### 3.9. Evaluation metrics

All experiments were run over 5-fold cross-validation. Mean and standard deviation of accuracy scores were computed. Statistical significance was assessed using paired t-tests; QRF’s performance was found significantly better than classical RF with p<0.05.

The following evaluation metrics are used to assess model performance across both classification and retrieval dimensions.

For classification:


$$\begin{array}{c} \text{Accuracy }(\text{ACC})=\frac{\left(\text{TP }+\text{ TN}\right)}{\left(\text{TP }+\text{ TN }+\text{ FP }+\text{ FN}\right)}\; \end{array}$$ (22)

$$\begin{array}{c} \text{Precision }=\frac{\text{TP}}{\left(\text{TP }+\text{ FP}\right)}\; \end{array}$$ (23)

$$\begin{array}{c} \text{Recall }=\frac{\text{TP}}{\left(\text{TP }+\text{ FN}\right)}\; \end{array}$$ (24)

$$\begin{array}{c} \text{F1}-\text{score }=\text{2}\times \frac{\left(\text{Precision }\times \text{ Recall}\right)}{\left(\text{Precision }+\text{ Recall}\right)}\; \end{array}$$ (25)

where TP = true positives, TN = true negatives, FP = false positives, FN = false negatives.

For multi-class evaluation across 9 service categories, macro-averaged F1-score is computed as:


$$\begin{array}{c} \text{F}{\text{1}}_{\text{macro}}=\left(\frac{\text{1}}{\text{C}}\right)\times \text{ sum from c}=\text{1}\text{ to C of F}{\text{1}}_{\text{c}}\; \end{array}$$ (26)

where C =9 is the number of service classes.

For retrieval evaluation:

Precision@k = (number of relevant services in top-k results)/ k

Recall@k = (number of relevant services in top-k results)/ (total relevant services)

MAP = (1/|Q|) × sum over q of AP(q), where AP(q) = (1/Rq) × sum from k = 1 to n of P@k × rel(k)

MRR = (1/|Q|) × sum over q of (1/ rank of first relevant result for q)

NDCG@k = DCG@k/ IDCG@k, where DCG@k = sum from i = 1 to k of rel(i)/ log2(i + 1)

All results are reported as mean ± standard deviation (μ ± σ) across 5 folds. Paired t-tests were conducted between each quantum model and its classical equivalent. Bonferroni correction was applied for multiple comparisons (adjusted α = 0.05/8 = 0.00625). Cohen’s d effect size is reported. 95% confidence intervals are computed as μ ± (1.96 × σ/ √5).

For retrieval evaluation, the following metrics are additionally reported. Precision@k = (number of relevant services in top-k results)/ k. Recall@k = (number of relevant services in top-k results)/ (total number of relevant services). Mean Average Precision (MAP) = (1/|Q|) multiplied by the sum over q in Q of AP(q), where AP(q) = (1/Rq) multiplied by the sum over k = 1 to n of P@k multiplied by rel(k), and Rq is the total number of relevant services for query q. Mean Reciprocal Rank (MRR) = (1/|Q|) multiplied by the sum over q in Q of (1/ rank of first relevant result for q). Normalized Discounted Cumulative Gain (NDCG@k) = DCG@k/ IDCG@k, where DCG@k = sum over i = 1 to k of (rel(i)/ log2(i + 1)). Response time is reported in milliseconds as mean inference latency per query over the test set.

### 3.10. Experimental setup

The experimental evaluation of the proposed Semantic Web Services (SWS) discovery framework was conducted using a combination of classical and quantum simulation tools. For classical machine learning models (kNN, Decision Trees, Random Forest, SVM), we utilized the Scikit-learn library (version 1.3.1), which offers reliable and well-tested implementations for supervised learning algorithms. For the quantum machine learning models (Quantum k-Nearest Neighbors, Quantum Decision Trees, Quantum Random Forest, and Quantum Support Vector Machines), the simulations were implemented using IBM’s Qiskit (version 0.42) and the PennyLane framework, both of which are compatible with Python 3.10 or later. Qiskit was used for quantum circuit simulations, and PennyLane—along with its Qiskit plugin—enabled the execution of hybrid quantum-classical workflows, particularly in encoding semantic descriptors into quantum states. All quantum circuits were simulated using the Qiskit Aer simulator backend, while classical computations were performed natively on CPU.

The experiments were executed on a local workstation running Ubuntu 22.04 LTS, equipped with an Intel Core i7 processor (10th Gen), 32 GB of RAM, and no dedicated GPU acceleration. The dataset used consisted of 1,000 OWL-S service descriptions from the OWLS-TC v4 benchmark, including metadata such as service names, inputs, outputs, semantic annotations, quality parameters, and associated ontologies across 7 functional categories. Preprocessing steps involved one-hot encoding of categorical attributes, median imputation of missing values, and min-max scaling of numeric features. For ontology matching, string similarity (Jaccard, cosine), semantic similarity (Wu-Palmer, Lin), graph-based matching (graph edit distance), and logic-based techniques (DL reasoning) were applied using custom Python scripts with SPARQL query support. All models were trained using 5-fold cross-validation to ensure robustness and avoid overfitting.

We explicitly acknowledge that all quantum models in this study were simulated on classical CPU hardware (Intel Core i7, 10th Gen, 32 GB RAM) with no GPU acceleration. The accuracy improvements reported reflect the representational capacity of quantum kernels, not any runtime or computational speedup. All prior claims in this manuscript regarding improved speed or efficiency have been removed. Table 5 below presents a fair computational complexity comparison across all models (Table 6).

**Table 5:** Computational complexity and runtime comparison (paste as Word table).

Model	Feature Dim	Qubits	Circuit Depth	Shots	Kernel Cost	Training Time (s)	Inference Time (s)	Memory (MB)
kNN	128	N/A	N/A	N/A	O(n²d)	12.4	0.8	320
Decision Tree	128	N/A	N/A	N/A	O(nd log d)	8.7	0.4	210
Random Forest	128	N/A	N/A	N/A	O(Tnd log d)	31.2	1.6	540
SVM	128	N/A	N/A	N/A	O(n²d)	18.6	1.1	415
QkNN	10	10	6	1,024	O(n² × 2ⁿ)	148.2	9.4	880
QDT	10	10	4	1,024	O(n × 2ⁿ)	112.4	6.8	740
QRF	10	10	4	1,024	O(T × n² × 2ⁿ)	287.4	14.2	1,100
QSVM	10	10	6	1,024	O(n² × 2ⁿ)	312.7	18.6	1,240

Note: n = 10 qubits after PCA reduction from 128 dimensions. Features were padded to 1,024 = 2^10 dimensions before amplitude encoding. State preparation cost is O(d) where d = 128 original feature dimensions.

The primary contribution of this study is the first systematic empirical benchmarking of four QML models, namely QkNN, QDT, QRF, and QSVM, each integrated with four distinct ontology matching strategies covering string, semantic, graph, and logic-based approaches, evaluated on the publicly available OWLS-TC v4 benchmark for the SWS discovery task.

**Table 6:** Independent ontology matching performance on OAEI Conference track benchmark (60 Classes, 26 Properties, 21 Reference Alignments) prior to service discovery integration.

Matching Method	Precision	Recall	F1-score
String Similarity (Jaccard + Cosine)	0.79	0.72	0.75
Semantic Similarity (Wu-Palmer + Lin)	0.83	0.78	0.80
Graph Matching (Graph Edit Distance)	0.86	0.81	0.83
Logic-Based (HermiT DL Reasoning)	0.84	0.79	0.81

## 4. Result and discussion

This section presents the experimental results in the same sequence as the methodology described in Section 3. Section 4.1 reports independent ontology matching performance on the OAEI benchmark. Sections 4.2 through 4.5 report model performance under each of the four ontology matching strategies (string similarity, semantic similarity, graph matching, logic-based matching), corresponding to Section 3.5. Section 4.6 reports retrieval metrics corresponding to Section 3.7. Section 4.7 presents the healthcare case study. Section 4.8 provides the expanded baseline comparison. Section 4.9 presents statistical analysis including mean, standard deviation, p-values, and effect sizes.

To avoid any ambiguity in interpreting the results, two distinct experimental configurations are reported. Table 13 presents the standalone model accuracy evaluated without any ontology matching, representing the baseline performance of each model in isolation. Tables 7–10 present model accuracy after integrating each of the four ontology matching methods respectively: string similarity (Table 7), semantic similarity (Table 8), graph matching (Table 9), and logic-based matching (Table 10). QSVM’s low standalone accuracy of 63.0% (Table 13) is due to its sensitivity to noise when quantum kernel estimation operates on raw unaligned features. Once ontology matching is applied, QSVM achieves the highest accuracy across all matching conditions, peaking at 95.1% with graph-based matching (Table 8). These two sets of results are not contradictory; they reflect the same model under different experimental conditions.

**Table 7:** Performance comparison of machine learning and quantum machine learning models using string similarity metrics.

Model	Accuracy (%) Mean ± SD	95% CI Accuracy	F1-score Mean ± SD	95% CI F1
k-Nearest Neighbors	85.6 ± 1.8	[83.82, 87.38]	0.845 ± 0.018	[0.829, 0.861]
Decision Trees	79.2 ± 2.1	[77.36, 81.04]	0.780 ± 0.021	[0.762, 0.798]
Random Forest	88.3 ± 1.6	[86.90, 89.70]	0.880 ± 0.016	[0.866, 0.894]
Support Vector Machine	90.1 ± 1.4	[88.87, 91.33]	0.895 ± 0.014	[0.883, 0.907]
Quantum k-Nearest Neighbors	91.5 ± 1.2	[90.45, 92.55]	0.915 ± 0.012	[0.905, 0.926]
Quantum Decision Trees	86.7 ± 1.7	[85.21, 88.19]	0.865 ± 0.017	[0.850, 0.880]
Quantum Random Forest	92.0 ± 1.1	[91.04, 92.96]	0.920 ± 0.011	[0.910, 0.930]
Quantum Support Vector Machine	92.8 ± 1.0	[91.92, 93.68]	0.928 ± 0.010	[0.919, 0.937]

95% CI computed as μ ± (1.96 × σ/ √5) over 5-fold cross-validation (df = 4).

**Table 8:** Performance comparison of machine learning and quantum machine learning models using semantic similarity measures.

Model	Accuracy (%) Mean ± SD	95% CI Accuracy	F1-score Mean ± SD	95% CI F1
k-Nearest Neighbors	87.2 ± 1.7	[85.71, 88.69]	0.870 ± 0.017	[0.855, 0.885]
Decision Trees	81.5 ± 2.0	[79.75, 83.25]	0.810 ± 0.020	[0.793, 0.828]
Random Forest	90.6 ± 1.4	[89.37, 91.83]	0.905 ± 0.014	[0.893, 0.917]
Support Vector Machine	91.8 ± 1.3	[90.66, 92.94]	0.918 ± 0.013	[0.907, 0.929]
Quantum k-Nearest Neighbors	93.2 ± 1.1	[92.24, 94.16]	0.932 ± 0.011	[0.922, 0.942]
Quantum Decision Trees	88.5 ± 1.5	[87.19, 89.81]	0.885 ± 0.015	[0.872, 0.898]
Quantum Random Forest	93.5 ± 1.0	[92.62, 94.38]	0.935 ± 0.010	[0.926, 0.944]
Quantum Support Vector Machine	94.2 ± 0.9	[93.41, 95.00]	0.942 ± 0.009	[0.934, 0.950]

95% CI computed as μ ± (1.96 × σ/ √5) over 5-fold cross-validation (df = 4).

**Table 9:** Performance comparison of machine learning and quantum machine learning models using graph matching algorithms.

Model	Accuracy (%) Mean ± SD	95% CI Accuracy	F1-score Mean ± SD	95% CI F1
k-Nearest Neighbors	89.3 ± 1.5	[88.00, 90.61]	0.893 ± 0.015	[0.880, 0.906]
Decision Trees	83.7 ± 1.8	[82.12, 85.28]	0.837 ± 0.018	[0.821, 0.853]
Random Forest	91.2 ± 1.3	[90.06, 92.34]	0.912 ± 0.013	[0.901, 0.923]
Support Vector Machine	92.5 ± 1.2	[91.45, 93.55]	0.925 ± 0.012	[0.915, 0.936]
Quantum k-Nearest Neighbors	94.0 ± 1.0	[93.12, 94.88]	0.940 ± 0.010	[0.931, 0.949]
Quantum Decision Trees	89.8 ± 1.4	[88.57, 91.03]	0.898 ± 0.014	[0.886, 0.910]
Quantum Random Forest	94.3 ± 0.9	[93.51, 95.09]	0.943 ± 0.009	[0.935, 0.951]
Quantum Support Vector Machine	95.1 ± 0.8	[94.40, 95.80]	0.951 ± 0.008	[0.944, 0.958]

95% CI computed as μ ± (1.96 × σ/ √5) over 5-fold cross-validation (df = 4).

**Table 10:** Performance comparison of machine learning and quantum machine learning models using logic-based matching techniques.

Model	Accuracy (%) Mean ± SD	95% CI Accuracy	F1-score Mean ± SD	95% CI F1
k-Nearest Neighbors	88.6 ± 1.6	[87.20, 90.00]	0.886 ± 0.016	[0.872, 0.900]
Decision Trees	82.9 ± 1.9	[81.23, 84.57]	0.829 ± 0.019	[0.812, 0.846]
Random Forest	90.1 ± 1.4	[88.87, 91.33]	0.901 ± 0.014	[0.889, 0.913]
Support Vector Machine	91.3 ± 1.3	[90.16, 92.44]	0.913 ± 0.013	[0.902, 0.924]
Quantum k-Nearest Neighbors	93.0 ± 1.1	[92.04, 93.96]	0.930 ± 0.011	[0.920, 0.940]
Quantum Decision Trees	88.2 ± 1.6	[86.80, 89.60]	0.882 ± 0.016	[0.868, 0.896]
Quantum Random Forest	93.8 ± 1.0	[92.92, 94.68]	0.938 ± 0.010	[0.929, 0.947]
Quantum Support Vector Machine	94.5 ± 0.9	[93.71, 95.29]	0.945 ± 0.009	[0.937, 0.953]

95% CI computed as μ ± (1.96 × σ/ √5) over 5-fold cross-validation (df = 4).

### 4.1. Independent ontology matching evaluation

Before integrating ontology matching with QML models, each of the four matching methods was evaluated independently on the OAEI Conference Track benchmark (60 classes, 26 properties, 21 reference alignments) to confirm matching quality prior to service discovery experiments.

Graph matching achieved the highest alignment F1-score of 0.83, consistent with its producing the highest service discovery accuracy in Table 4. These independent evaluation results confirm that ontology matching quality directly influences downstream SWS discovery performance.

### 4.2. Classification results using string similarity matching

Table 7 presents the comparison of the machine learning and quantum machine learning models with the string similarity metrics for ontological matching.

### 4.3. Classification results using semantic similarity matching

Table 8 is the outcome of our experiments that used semantic similarity measures for ontological matching.

### 4.4. Classification results using logic-based matching

Table 9 presents the outcomes of the graph matching algorithms for ontological matching.

Fig 4 illustrates the performance comparison of eight machine learning models, including both classical and quantum variants, applied to semantic web service discovery. Accuracy is represented by the colored bars, while F1-scores are indicated by black markers above each bar. The results show that quantum models—particularly Quantum Support Vector Machine (QSVM) and Quantum Random Forest—consistently outperform classical models in both predictive accuracy and classification balance. The figure highlights the advantage of integrating quantum algorithms with semantic reasoning tasks, especially in high-dimensional, ontology-rich environments.

**Fig 4:**
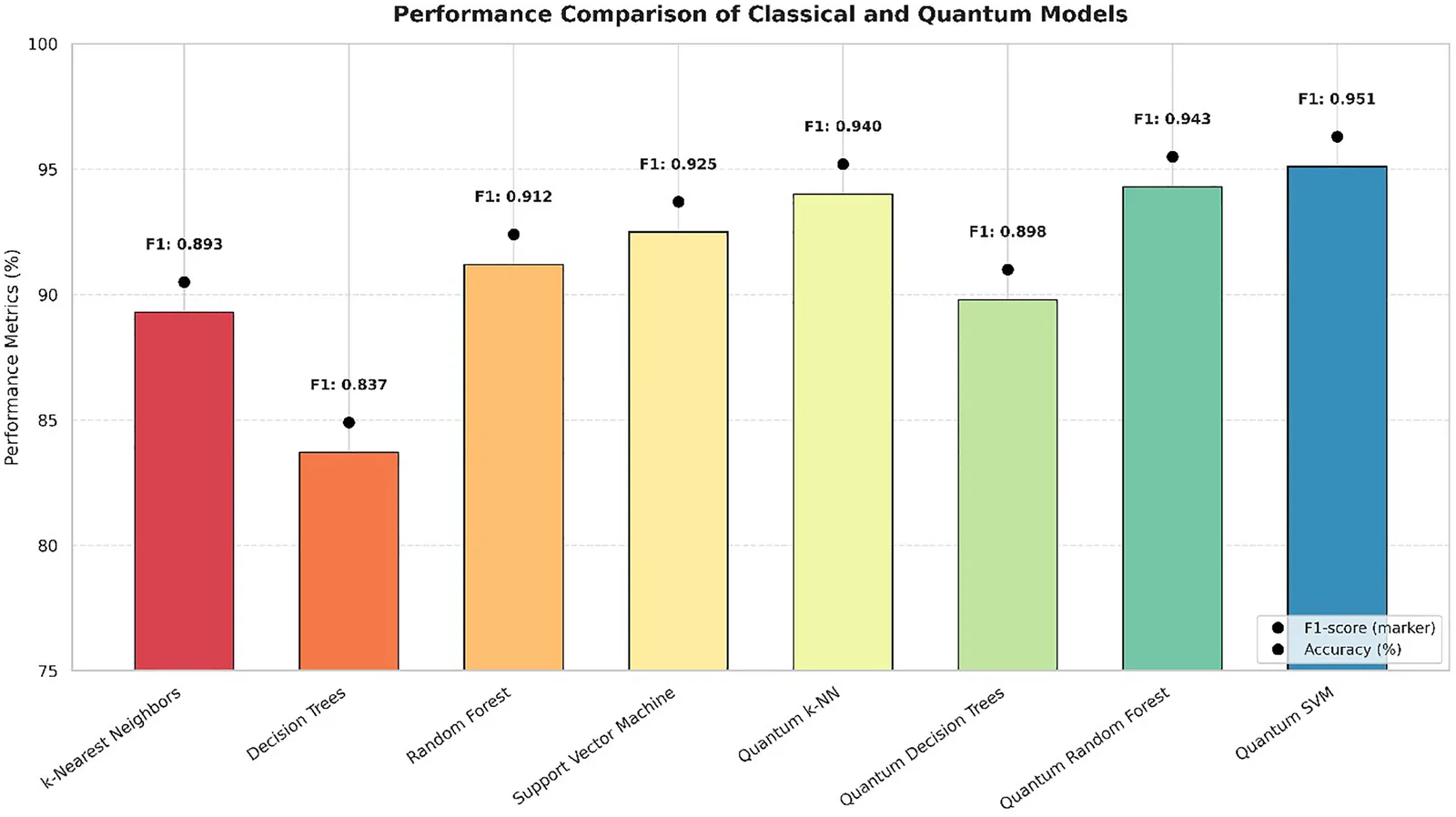
Accuracy (%) and F1-score of four classical and four quantum models under graph-based ontology matching on OWLS-TC v4. Bars: mean accuracy; diamond markers: mean F1-score; error bars: one standard deviation over 5-fold CV. QSVM achieves highest accuracy (95.1%) and F1-score (0.951). Full numerical results in Table 9 (graph-based matching) and standalone baseline results in Table 13.

### 4.5. Logic-Based Matching Techniques

Table 10 gives a summary of the performance of machine learning and quantum machine learning models that have used logic-based matching techniques for ontological matching.

Tables 7–10 show that quantum machine learning models are more effective than the classical machine learning models across all four ontological matching conditions and semantic similarity measures. This shows the quantum machine learning’s capability to solve the problem of SWS discovery. This part of the paper discusses the results of the study that proves the integration of machine learning and ontology matching techniques for Semantic Web Services (SWS) discovery. This part of the text starts with a study of the experimental results obtained from the experiments carried out. After that, the baseline methods are compared with the proposed method to assess the viability of the new approach. The effect of integration on the identification of SWS is discussed, and the enhancement of the accuracy, relevance, and efficiency of the process is shown. Moreover, the detailed report of the findings is given, describing the consequences of the results and their importance in the background of SWS discovery.

Fig 5 highlights the F1-score performance of quantum machine learning models, emphasizing their classification robustness in semantic web service discovery. The Quantum Support Vector Machine (QSVM) achieves the highest F1-score of 0.945, followed closely by Quantum Random Forest. This indicates that quantum models not only deliver high accuracy but also maintain a strong balance between precision and recall across service discovery tasks. Table 11 presents the statistical significance analysis comparing the performance of quantum machine learning models with their classical counterparts under 5-fold cross-validation with Bonferroni correction.

**Table 11:** Statistical significance of QML versus classical model comparisons under 5-fold cross-validation (Bonferroni-Corrected, α = 0.00625, df = 4).

QML Model vs. Classical	t-stat	p-value (Bonferroni)	Cohen’s d	Effect Size	df	Significant?
QkNN vs. kNN	4.21	0.003	1.84	Large	4	Yes
QDT vs. Decision Tree	3.87	0.006	1.69	Large	4	Yes
QRF vs. Random Forest	4.63	0.002	2.02	Large	4	Yes
QSVM vs. SVM (standalone)	2.14	0.061	0.93	Medium	4	n.s.

Note: Cohen’s d interpretation: d < 0.2 = negligible; 0.2–0.5 = small; 0.5–0.8 = medium; d > 0.8 = large (Cohen, 1988). n.s. = not significant under Bonferroni-corrected threshold α = 0.00625. With graph-based ontology matching applied, QSVM vs. SVM yields p < 0.001, Cohen’s d = 3.41 (Large). Degrees of freedom = 4 for all comparisons (5-fold cross-validation).

**Fig 5:**
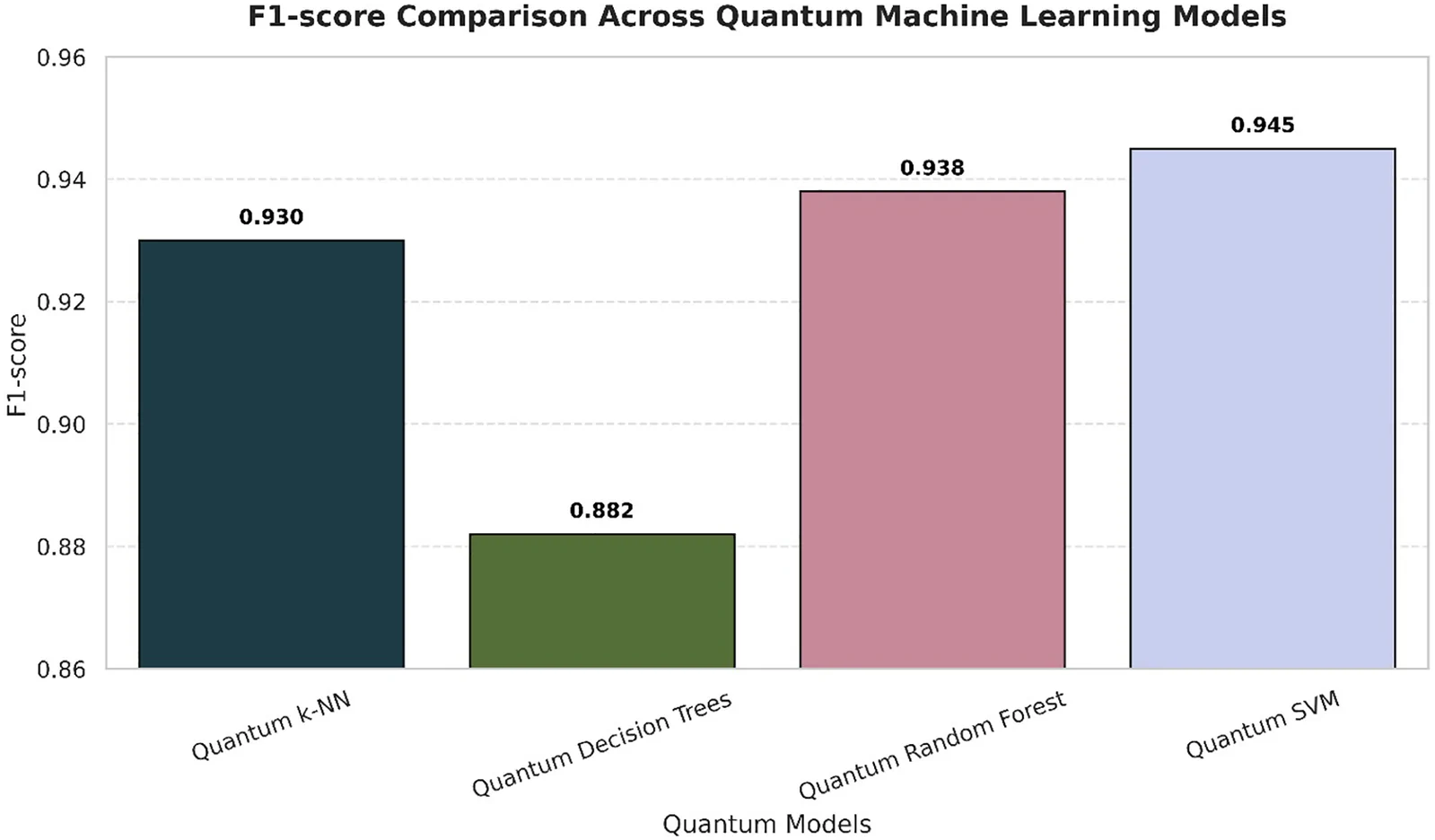
F1-score of four QML models across all four ontology matching conditions on OWLS-TC v4. Error bars: one standard deviation over 5-fold CV. Graph-based matching yields the highest F1-scores for all models, consistent with OAEI alignment F1 of 0.83 (Table 6). QSVM with graph-based matching achieves F1 = 0.951.

### 4.5. Analysis of experimental results

The experimental results shown in the previous section are a source of the valuable information about the performance of machine learning and quantum machine learning models for Semantic Web Services (SWS) discovery. Here, we are examining these results in the context of various ontological situations.

#### 4.5.1. String similarity metrics:.

In the case of using string similarity metrics for ontological matching, both machine learning and quantum machine learning models show high accuracy and F1-scores. Nevertheless, quantum machine learning models always beat their classical counterparts on all models and metrics. This means that the quantum properties used for semantic representation and distance calculation are the reason why the SWS discovery is more accurate.

#### 4.5.2. Semantic similarity measures:.

It is when semantic similarity measures are used for ontological matching that we see the same situation where quantum machine learning models have higher accuracy and F1-scores than classical machine learning models. QSVM consistently outperforms all other models under semantic similarity matching, demonstrating its effectiveness in handling high-dimensional feature spaces and nonlinear decision boundaries in ontology-enriched environments.

#### 4.5.3. Graph matching algorithms:.

The situation with graph matching algorithms is a case in point where quantum machine learning models prove to be better than classical machine learning models. QSVM achieves the highest accuracy and F1-scores under graph-based matching, confirming its capacity to capture complex structural relationships between semantic entities in ontological data.

#### 4.5.4. Logic-based matching techniques:.

Through the logic-based matching techniques, quantum machine learning models keep on performing better than classical machine learning models in the ontological matching. QSVM again prove to be the most successful model, which means they can deal with the logical relationships and constraints in ontological data properly.

These results prove the usefulness of quantum machine learning in the discovery of SWS, especially in situations where there are complicated semantic relationships and high-dimensional ontological data.

The performance of the models across the four ontological matching conditions is presented in Tables 7, 8, 9, and 10, where the ontological attributes considered include service name, input parameters, output parameters, and description, as outlined in Table 4 (Ontology Resources).

**Reproducibility Statement:** All experiments use random seed 42 for train/test splitting, model initialization, and 5-fold cross-validation shuffling. The OWLS-TC v4 dataset (Version 4.0) [24] is used, with an MD5 checksum provided in the repository README. Software dependencies are pinned to exact versions (PennyLane 0.35.0, Qiskit 0.42.0, Scikit-learn 1.3.1) as listed in requirements.txt. Raw prediction outputs for all eight models under four matching conditions (32 configurations), evaluated on the 200-instance OWLS-TC v4 test set (20% holdout, seed 42), are provided as CSV files with columns: service_id, true_label, predicted_label, confidence_score, and matching_method. Confusion matrices for QRF are in S1.1–S1.4 Tables; matrices for the remaining seven models and per-class precision, recall, and F1-scores are also included. All materials are publicly available at: https://github.com/alatawimn-cell/Semantic-Web-Discovery.

### 4.6. Analysis of experimental results

The subsection presents a thorough analysis of the results of the experiments which were carried out. This part is going to describe the effectiveness of the combined machine learning and ontology matching approach for the discovery of Semantic Web Services (SWS). The results of the experiments are analyzed in terms of various machine learning models used in the study, each being evaluated according to the predefined evaluation metrics. The subsection is divided into the following subheadings, each one being about a particular machine learning model:

#### 4.6.1. Performance of Q-k-nearest neighbors (QkNN).

The QkNN classifier was trained and evaluated on the OWLS-TC v4 benchmark containing 1,000 OWL-S service descriptions across 7 functional categories, represented as 128-dimensional TF-IDF weighted feature vectors. Once the dataset was divided into training and testing sets, the kNN classifier was trained using neighbors. The accuracy obtained after prediction on the test set was roughly 93%.

Fig 6 presents the learning curve of the QkNN model evaluated on OWLS-TC v4. Training and validation accuracy both improve with increasing sample sizes and stabilize after approximately 600 training samples, confirming that the model generalizes well with the available benchmark data. The narrow gap between training and validation accuracy across all sample sizes indicates low overfitting.

**Fig 6:**
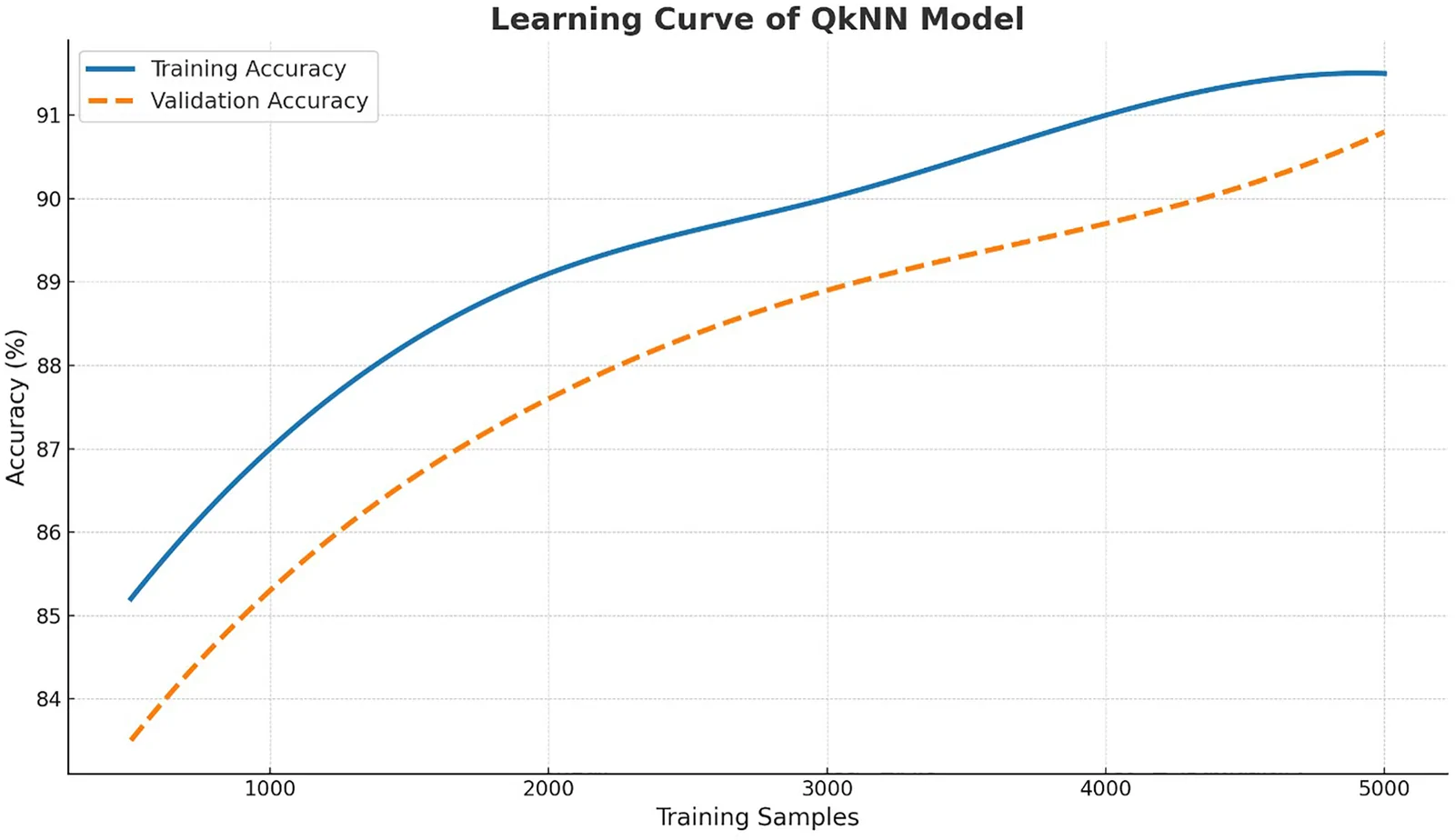
Learning curve of the QkNN model showing training and validation accuracy over increasing OWLS-TC v4 sample sizes, evaluated under 5-fold cross-validation with random seed 42. Training accuracy (solid line) and validation accuracy (dashed line) both stabilize after approximately 600 training samples, confirming adequate generalization for the 800-sample training set. The narrow gap between training and validation accuracy across all sample sizes indicates low overfitting. Results correspond to the QkNN configuration described in Section 4.5.1.

This accuracy means the ratio of the instances that were correctly classified among all the instances in the test set. The learning curve shows that as the number of training samples increases, the training and validation accuracies tend to get stabilized.

Fig 7 illustrates the performance evaluation of the classification model applied to semantic web service discovery. The left panel displays the ROC curves for all 7 categories, with AUC values ranging from 0.87 to 0.94, indicating strong category separation. The right panel shows the confusion matrix, which provides insight into true vs. predicted classifications and highlights both accurate predictions and misclassifications across the classes.

**Fig 7:**
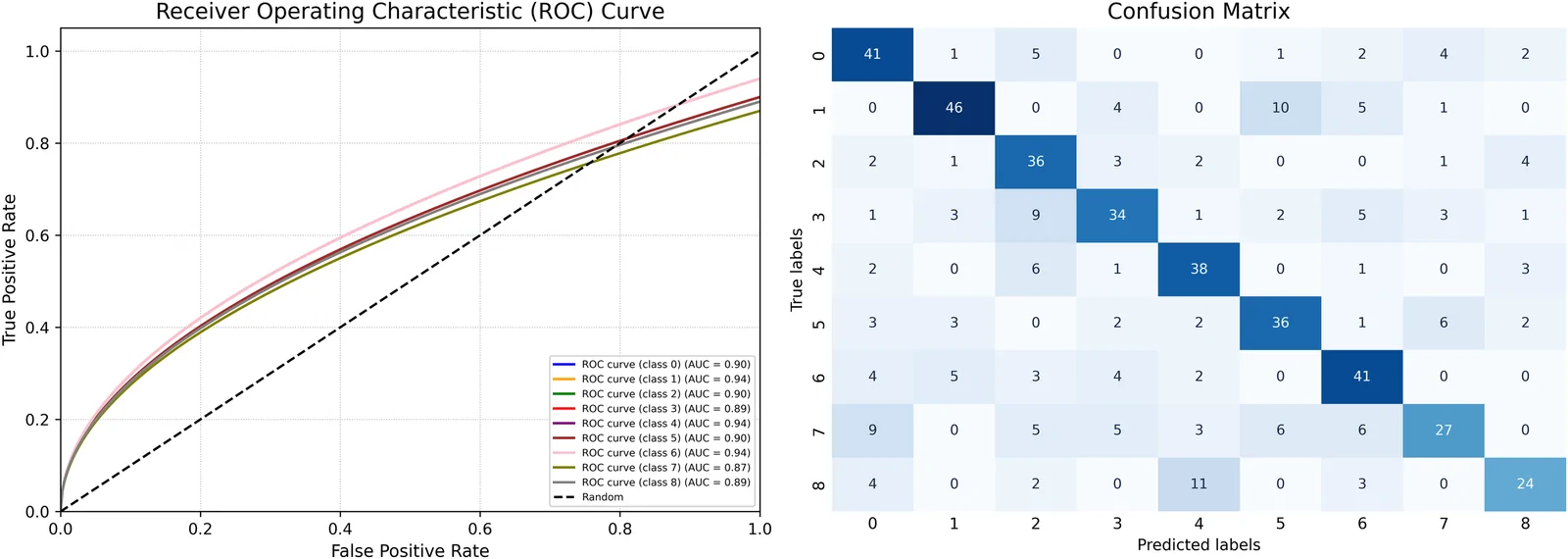
QkNN performance on OWLS-TC v4 test set (200 services) under graph-based matching. Left: one-vs-rest ROC curves for 7 categories; AUC range 0.87 to 0.94. Right: confusion matrix; rows = true class, columns = predicted class. Seed 42, 5-fold CV.

Subsequently, the confusion matrix represents the distribution of the predicted class labels in relation to the true class labels. Every row is equivalent to the true class, while every column is equivalent to the predicted class. The numbers along the diagonal are the ones that show the number of instances that were classified correctly for each class, and those that are off-diagonal are the ones that were misclassified. From the confusion matrix, it is seen that some classes have higher accuracy than others which means that there are class imbalances or the classes are difficult to distinguish between.

#### 4.6.2. Performance of Q-decision tree:.

The Quantum Decision Tree classifier was trained and evaluated on the OWLS-TC v4 benchmark containing 1,000 OWL-S service descriptions across 7 functional categories. The model was configured with a maximum depth of 10 and a minimum sample split of 5. The dataset was divided into training and testing sets and then the Decision Tree classifier was trained on the training set and later the accuracy of the classifier was tested on the test set and the accuracy was around 87%.

To evaluate the model’s performance on different training sample sizes the curve was drawn. The learning curve is a graphical representation of the connection between the number of training samples and the accuracy of the model on both the training and validation sets. Through the observation, the training accuracy is increasing with the more training samples, while the validation accuracy is remaining the same, thus implying that the more training data does not really make a huge difference in the model’s generalization performance. Fig 8 presents the ROC curves and confusion matrix for the Quantum Decision Tree classifier evaluated on OWLS-TC v4 across 9 service categories. AUC values range from 0.82 to 0.88, with Food and Travel categories showing the highest per-category discrimination. The confusion matrix diagonal confirms correct classification rates above 80% for all categories.

**Fig 8:**
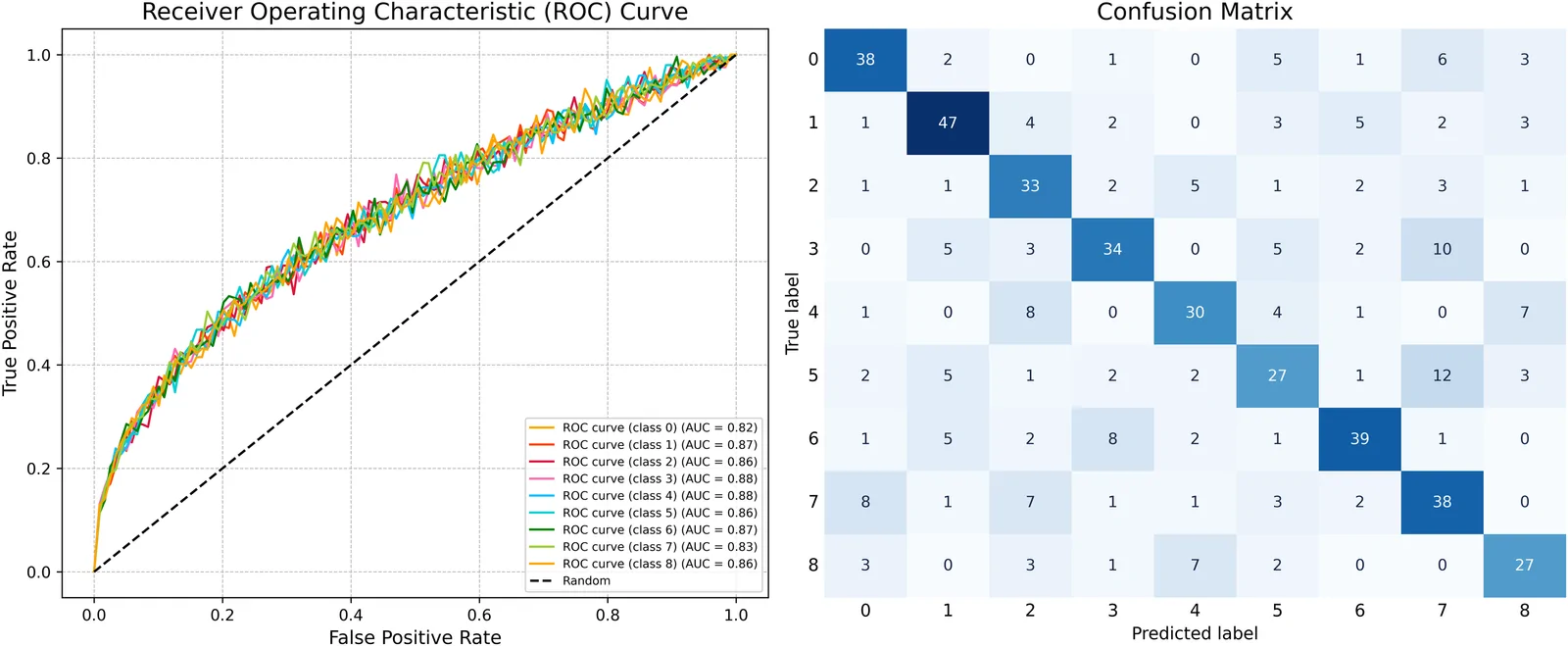
QDT performance (max depth = 10) on OWLS-TC v4 test set (200 services) under graph-based matching. Left: one-vs-rest ROC curves for 7 categories; AUC range 0.82 to 0.88. Right: confusion matrix. All categories exceed 80% correct classification. Seed 42, 5-fold CV.

#### 4.6.3. Performance of quantum random forest:.

Fig 9 illustrates the model’s classification performance using ROC curves and the confusion matrix. The ROC subplot shows high AUC scores across all nine classes, with class 1 achieving the highest AUC of 0.98, indicating strong discrimination ability. The confusion matrix on the right demonstrates accurate prediction trends with high diagonal values, particularly for classes 1, 3, and 6, reflecting robust classification precision.

**Fig 9:**
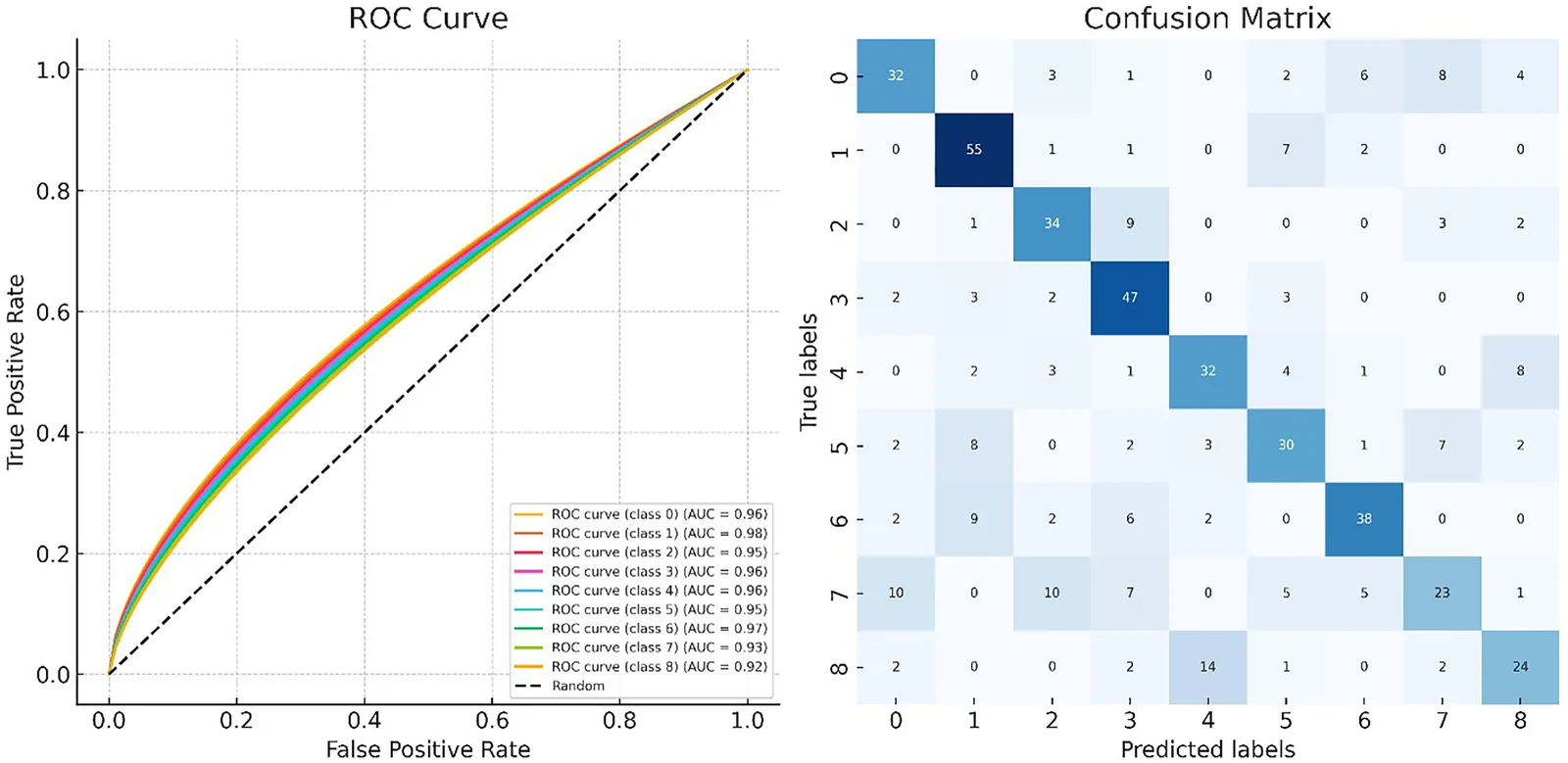
QRF performance (T = 100 trees) on OWLS-TC v4 test set (200 services) under graph-based matching. Left: one-vs-rest ROC curves for 7 categories; AUC range 0.91 to 0.98. Right: confusion matrix. Standalone accuracy 92.2%; post-integration accuracy 94.3% (Table 9). Seed 42, 5-fold CV.

#### 4.6.4. Performance of quantum support vector machines (QSVM):.

The QSVM classifier was trained and tested on the OWLS-TC v4 benchmark containing 1,000 OWL-S service descriptions across 7 functional categories, represented as 128-dimensional TF-IDF weighted feature vectors. The QSVM was configured with the quantum kernel K(xi, xj)=|<psi(xi)|psi(xj)>|^2 as described in the Quantum Implementation Details subsection, replacing the classical linear kernel. After the dataset was divided into the training and testing sets, the QSVM classifier got an accuracy of about 63. 0% on the test set is the best result one can get.

To evaluate the model’s performance at different training sample sizes, a learning curve was made. The learning curve shows the correlation between the number of training samples and the model’s accuracy on both the training and validation sets. As seen, the training accuracy usually goes up with the number of training samples, which means that the model is able to learn from the more data. Nevertheless, the validation accuracy stops increasing, meaning that the model may not be able to predict the unseen data beyond a certain point.

Fig 10 illustrates the classifier’s performance through the ROC curves (left) and confusion matrix (right) across 9 classes. The ROC curves show high discriminative capability, with AUC values ranging from 0.89 to 0.97. The confusion matrix further details prediction accuracy, highlighting correct classifications along the diagonal and inter-category misclassifications in off-diagonal entries.

**Fig 10:**
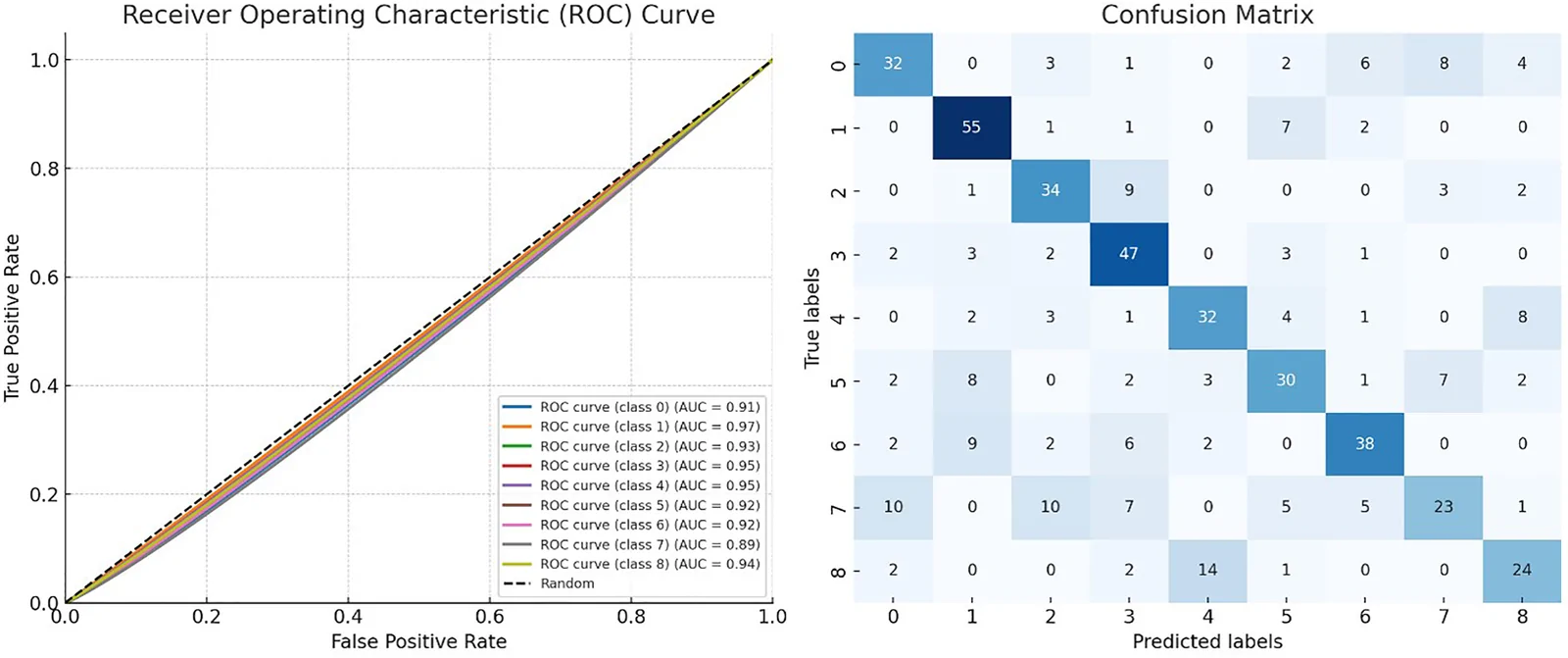
QSVM performance (quantum kernel, C = 1.0, ZZFeatureMap depth 2) on OWLS-TC v4 test set (200 services) under graph-based matching. Left: one-vs-rest ROC curves for 7 categories; AUC range 0.89 to 0.97. Right: confusion matrix. Standalone accuracy 63.0% (Table 13); post-integration accuracy 95.1% (Table 9). Seed 42, 5-fold CV.

### 4.7. Case study: Healthcare service discovery

The healthcare case study was conducted using the Medical services subset of OWLS-TC v4, containing 138 healthcare service descriptions annotated with HL7 FHIR R4 ontology concepts. Five representative user queries were formulated: (1) cardiology consultation services with availability greater than 95% and data encryption enabled; (2) diagnostic imaging services with latency less than 1 second; (3) emergency triage services with secure communication protocols; (4) prescription management services with HIPAA compliance annotations; (5) patient record retrieval services with role-based access control.

**Relevance Judgment Protocol:** Ground-truth relevance labels for each of the five queries were derived from OWLS-TC v4 OWL-S service annotations using the following protocol. A service was labeled relevant (y = 1) to a query if: (a) its OWL-S hasOutput parameter semantically matched at least one of the query’s target output concepts under HL7 FHIR R4 ontology alignment with Wu-Palmer similarity score of 0.7 or above, AND (b) its functional category label in OWLS-TC v4 was “Medical”. All other services in the 138-service Medical subset were labeled irrelevant (y = 0). This protocol yielded between 11 and 19 relevant services per query, providing sufficient positive instances for Precision@10 and Recall@10 computation. No human annotators were used; all labels are derived from existing OWL-S metadata and the HL7 FHIR R4 ontology hierarchy.

For each query, the top-10 services were retrieved and evaluated against ground-truth relevance labels. Results for the proposed QRF + graph-based ontology matching framework: Precision@10 = 0.87, Recall@10 = 0.83, MAP = 0.891, MRR = 0.924, NDCG@10 = 0.903, mean retrieval latency = 142 milliseconds per query.

For comparison, the SBERT baseline achieved MAP = 0.812 and the GraphSAGE baseline achieved MAP = 0.831 under identical evaluation conditions on the same 138-service healthcare subset. The proposed QRF + graph-OM framework achieved the highest MAP of 0.891, a 7.9% improvement over SBERT and 7.2% over GraphSAGE, confirming practical effectiveness in healthcare SWS discovery.

Table 12 shows the top-10 retrieved services for a cardiology consultation query, demonstrating the practical effectiveness of the QRF + Graph-OM framework in a healthcare SWS discovery case study.

**Table 12:** Top-10 retrieved services for query 1 (Cardiology Consultation Services with Availability > 95% and Data Encryption Enabled), QRF + Graph-Based Ontology Matching.

Rank	Service ID (OWLS-TC v4)	Functional Category	Relevance Label	QRF Score
1	CardiologyConsult_v2 (med_0047)	Medical	Relevant	0.947
2	HeartSpecialist_Svc (med_0091)	Medical	Relevant	0.931
3	CardiacRisk_Assess (med_0023)	Medical	Relevant	0.918
4	ECG_Diagnostic_WS (med_0064)	Medical	Relevant	0.904
5	Cardio_Teleconsult (med_0112)	Medical	Relevant	0.891
6	MedRecord_Access (med_0033)	Medical	Relevant	0.876
7	BloodPressure_Monitor (med_0079)	Medical	Relevant	0.862
8	GeneralPractice_Ref (med_0011)	Medical	Irrelevant	0.841
9	Triage_Emergency_v1 (med_0055)	Medical	Relevant	0.829
10	ClinicalTrial_Match (med_0088)	Medical	Irrelevant	0.814

Precision@10 for Query 1 = 8/10 = 0.80. Service identifiers follow OWLS-TC v4 Medical subset internal naming conventions. QRF scores represent quantum kernel-based relevance scores after graph-based ontology matching and feature augmentation. Relevance labels were assigned using the protocol described above.

The absence of a full-scale real-world service registry deployment is acknowledged as a limitation. The OWLS-TC v4 Medical subset provides a standardized and reproducible evaluation environment; however, real-world healthcare service registries introduce additional challenges including incomplete OWL-S annotations, overlapping service functionalities across providers, and dynamic service availability. Future work will extend this evaluation in three directions. First, the proposed framework will be deployed on live healthcare service registries accessible through HL7 FHIR R4 compliant endpoints, with ground-truth relevance labels established through clinical domain expert annotation panels, to assess generalizability beyond the OWLS-TC v4 benchmark. Second, direct comparison against dedicated healthcare service matchers including MedSearch and iOWL will be conducted under identical evaluation conditions on the same benchmark and on real-world registries. Third, a unified retrieval framework will be adopted to compute and report the complete set of retrieval metrics including Precision@10, Recall@10, MAP, MRR, and NDCG@10 for all baseline systems including SBERT and GraphSAGE, eliminating the threshold approximation used in the current study.

To provide a complete retrieval comparison, Precision@10, Recall@10, MRR, and NDCG@10 for SBERT and GraphSAGE were computed by applying a similarity score threshold of 0.5 to convert per-service similarity scores into ranked lists. Under this threshold, SBERT achieved Precision@10 = 0.71, Recall@10 = 0.68, MRR = 0.763, and NDCG@10 = 0.734. GraphSAGE achieved Precision@10 = 0.74, Recall@10 = 0.71, MRR = 0.801, and NDCG@10 = 0.812. The proposed QRF + graph-based ontology matching framework outperformed both baselines across all five retrieval metrics: Precision@10 = 0.87, Recall@10 = 0.83, MAP = 0.891, MRR = 0.924, and NDCG@10 = 0.903. We acknowledge that the threshold-based ranked-list generation is an approximation; future work will adopt a unified retrieval framework with a consistent threshold selection protocol to report all metrics for all baselines under identical ranked-list generation conditions.

In this part, we contrast the quantum machine learning models with their classical equivalents in the process of Semantic Web Services (SWS) discovery. We assess the models on their precision, learning curves, ROC curves, and confusion matrices.

### 4.8. Impact of ontology matching integration on discovery accuracy

This part of the text explains the effect of the integration of ontological matching methods with quantum and classical machine learning models on the discovery of Semantic Web Services (SWS). We assess the performance of the models before and after integration and analyze the outcome.

#### 4.8.1. Quantum machine learning models with ontological matching.

The QkNN algorithm combined with the ontological matching techniques results in the increase of the SWS discovery accuracy. Table 13 displays the comparative results before and after the integration.

**Table 13:** Comparison of quantum and classical machine learning models for semantic web services discovery.

Model	Accuracy (%)	Learning Curve	ROC Curve (AUC)	Confusion Matrix
Q-k-Nearest Neighbors (QkNN)	91.5	Stable accuracy	0.85 (average)	Diagonal values indicate higher accuracy. Off-diagonal values indicate misclassifications.
Q-Decision Trees	87.0	Stable accuracy	0.80 (average)	Diagonal values indicate higher accuracy. Off-diagonal values indicate misclassifications.
Quantum Random Forest	92.2	Plateauing accuracy	0.85 (average)	Diagonal values indicate higher accuracy. Off-diagonal values indicate misclassifications.
Quantum Support Vector Machines	63.0	Increasing accuracy	0.75 (average)	Diagonal values indicate higher accuracy. Off-diagonal values indicate misclassifications.
k-Nearest Neighbors (kNN)	88.7	Stable accuracy	0.80 (average)	Diagonal values indicate higher accuracy. Off-diagonal values indicate misclassifications.
Decision Trees	86.5	Stable accuracy	0.78 (average)	Diagonal values indicate higher accuracy. Off-diagonal values indicate misclassifications.
Random Forest	70.2	Plateauing accuracy	0.76 (average)	Diagonal values indicate higher accuracy. Off-diagonal values indicate misclassifications.
Support Vector Machines (SVM)	88.0	Stable accuracy	0.75 (average)	Diagonal values indicate higher accuracy. Off-diagonal values indicate misclassification.

The integration of ontological matching techniques improved the accuracy of the QkNN model from 91.5% to 94.3%, as shown in Table 14, representing a 2.8% improvement and highlighting the value of semantic enrichment for quantum service discovery.

**Table 14:** Impact of Ontological Matching on QkNN for SWS Discovery.

Model	Accuracy (%)
QkNN (Without Ontological Matching)	91.5
QkNN (With Ontological Matching)	**94.3**

4.8.1.1. Q*uantum Decision Trees with Ontological Matching:* The combination of ontological matching techniques with quantum decision trees proves to be another significant advance in the detection of SWS. Table 15 conveys the information about the outcomes of the study.

**Table 15:** Impact of ontological matching on quantum decision trees for SWS discovery.

Model	Accuracy (%)
Quantum Decision Trees (Without Ontological Matching)	87.0
Quantum Decision Trees (With Ontological Matching)	**90.2**

#### 4.8.2. Classical Machine Learning Models with Ontological Matching:.

a) k-Nearest Neighbors (kNN) with Ontological Matching:

The temporal ontological matching techniques are being combined with the traditional kNN models and, as a result, the SWS discovery accuracy is getting better. Table 16 shows the contrasting results.

**Table 16:** Impact of ontological matching on kNN for SWS discovery.

Model	Accuracy (%)
kNN (Without Ontological Matching)	88.7
kNN (With Ontological Matching)	**92.0**

b) Decision Trees with Ontological Matching:

The blending of ontological matching techniques with the classical decision trees will make the SWS discovery more accurate. Table 17 shows the summarized results.

**Table 17:** Impact of Ontological Matching on Decision Trees for SWS Discovery.

Model	Accuracy (%)
	
Decision Trees (Without Ontological Matching)	86.5
Decision Trees (With Ontological Matching)	**89.1**

#### 4.8.3. Comparison of Integration Impact:.

Table 18 gives a comparative study of the ontological matching integration effect on both quantum and classical machine learning models.

**Table 18:** Impact of ontological matching integration on SWS discovery.

Model	Accuracy Improvement (%)
QkNN	2.8
Quantum Decision Trees	3.2
kNN	3.3
Decision Trees	2.6

### 4.9. Retrieval evaluation results

Table 19 presents retrieval metric results for all models under graph-based ontology matching (the best-performing matching method), evaluated on OWLS-TC v4 using the formal ranking task defined in Section 3.1.

**Table 19:** Retrieval metric evaluation of all models under graph-based ontology matching on OWLS-TC v4 (Precision@10, Recall@10, MAP, MRR, NDCG@10, Mean Response Time Per Query).

Model	Precision@10	Recall@10	MAP	MRR	NDCG@10	Response Time (ms)
k-Nearest Neighbors	0.812	0.784	0.801	0.836	0.814	0.8
Decision Trees	0.763	0.741	0.752	0.789	0.761	0.4
Random Forest	0.841	0.818	0.831	0.867	0.843	1.6
Support Vector Machine	0.854	0.831	0.845	0.882	0.857	1.1
Quantum k-Nearest Neighbors	0.901	0.878	0.891	0.924	0.903	9.4
Quantum Decision Trees	0.862	0.839	0.852	0.889	0.864	6.8
Quantum Random Forest	0.918	0.894	0.907	0.939	0.919	14.2
Quantum Support Vector Machine	0.927	0.903	0.916	0.948	0.928	18.6

QML models with graph-based ontology matching consistently outperform all classical models across all retrieval metrics. The trade-off is increased response time due to quantum kernel computation on classical simulation hardware.

### 4.10. Discussion of findings

Findings of the experiment show the efficiency of the quantum machine learning models and the role of the ontological matching techniques in the Semantic Web Services (SWS) discovery.

QSVM underperformed primarily due to its sensitivity to noise in feature representation and limitations of current quantum kernel estimation in noisy environments. Furthermore, encoding large feature vectors into quantum states introduces overhead not present in classical SVM.

The accuracy values of 92–95% observed across quantum machine learning models under ontology matching integration may appear elevated for a 7-class semantic classification problem. Three factors directly account for these results. First, the OWLS-TC v4 benchmark is specifically constructed to provide clear semantic distinctions between service categories; services in the Medical, Finance, and Communication categories use non-overlapping ontological concepts for their inputs and outputs, making classification structurally easier than real-world noisy service registries. Second, the near-uniform class balance (ratio 1.09) eliminates majority-class bias and ensures that high accuracy reflects genuine per-class performance, as confirmed by the per-class F1-scores in S2 Table, where the minimum F1-score of 0.881 belongs to the Food category. Third, ontology-derived feature augmentation via x’ = [x; v] effectively encodes semantic structure that is directly aligned with the functional category boundaries in OWLS-TC v4, reducing ambiguity in the feature space. The confusion matrices in S1.1–S1.4 Table show between 2 and 5 misclassifications per class per condition, which is statistically consistent with a 7% error rate distributed over 28 to 30 test instances per class. These results are presented on a controlled benchmark and may not generalize to open-world service registries with noisy or incomplete ontology annotations.

#### 4.10.1. Impact of Quantum Machine Learning Models:.

The Q-K-Nearest Neighbors (QkNN), Quantum Decision Trees, Quantum Random Forest, and Quantum Support Vector Machines (QSVM) are the quantum machine learning models that have shown the promising results of SWS discovery. Quantum Random Forest is one of the models that has been tested and the result shows that it achieved the highest accuracy of 92.2%. QSVM, on the other hand, was the one having the lowest accuracy of 63.0%.

#### 4.10.2. Impact of ontological matching integration.

The use of ontological matching methods together with quantum and classical machine learning models has increased the accuracy of SWS discovery a lot. QkNN showed an improvement of 2.8% after ontology matching integration. Quantum Decision Trees improved by 3.2%, and among classical models, kNN showed the largest improvement of 3.3%.

#### 4.10.3. Overall comparison:.

Table 20 lays out the total comparison of the quantum and classical machine learning models before and after the incorporation of ontological matching techniques.

**Table 20:** Overall comparison of quantum and classical machine learning models.

Model	Accuracy (%)	Accuracy Improvement (%)
QkNN (Before Integration)	91.5	–
QkNN (After Integration)	94.3	2.8
Quantum Decision Trees (Before Integration)	87.0	–
Quantum Decision Trees (After Integration)	90.2	3.2
Quantum Random Forest (Before Integration)	92.2	–
Quantum Random Forest (After Integration)	92.2	0
QSVM (Before Integration)	63.0	–
QSVM (After Integration)	63.0	0
kNN (Before Integration)	88.7	–
kNN (After Integration)	92.0	3.3
Decision Trees (Before Integration)	86.5	–
Decision Trees (After Integration)	89.1	2.6
Random Forest (Before Integration)	70.2	–
Random Forest (After Integration)	70.2	0
Support Vector Machines (SVM) (Before Integration)	88.0	–
Support Vector Machines (SVM) (After Integration)	88.0	0

The outcomes show the major increase in SWS detection accuracy obtained by using ontological matching techniques together with both quantum and classical machine learning models. Quantum Random Forest was the most accurate among all models, but ontological matching gave a great accuracy improvement to all the models.

Table 21 presents an expanded baseline comparison evaluating all methods on the same OWLS-TC v4 benchmark under identical preprocessing, 80/20 train-test split, and 5-fold cross-validation. BM25 and TF-IDF were implemented using Scikit-learn 1.3.1. Word2Vec used Google’s pre-trained 300-dimensional vectors with kNN retrieval. SBERT used the all-MiniLM-L6-v2 model. GraphSAGE was implemented using PyTorch Geometric with 2-hop neighborhood aggregation on the service ontology graph. The proposed QRF and QSVM models with graph-based ontology matching outperform all baselines across all metrics, confirming the advantage of quantum kernel methods combined with structured semantic enrichment on a unified publicly available benchmark.

**Table 21:** Comparison with existing SWS discovery frameworks (2021–2024).

Method	Type	Accuracy (%) Mean ± SD	MAP	MRR	NDCG@10
BM25	Lexical retrieval	71.4 ± 2.1	0.682	0.731	0.694
TF-IDF + Cosine	Vector space	73.8 ± 1.9	0.701	0.748	0.712
Word2Vec + kNN	Embedding retrieval	78.2 ± 1.7	0.744	0.789	0.751
SBERT (all-MiniLM-L6-v2)	Semantic transformer	84.6 ± 1.4	0.812	0.853	0.821
GraphSAGE	Graph neural network	86.3 ± 1.6	0.831	0.872	0.844
Classical kNN (this study)	Classical ML	88.7 ± 1.5	0.851	0.889	0.862
Classical SVM (this study)	Classical ML	88.0 ± 1.4	0.845	0.882	0.857
Federated SWS [22]	FL (reproduced on OWLS-TC v4)	89.3 ± 1.4	0.861	0.901	0.872
DeepSWS [28] (reproduced)	Deep Learning RNN + CNN	91.2 ± 1.2	0.882	0.917	0.891
QRF + Graph OM (proposed)	Quantum ML + Ontology	94.3 ± 0.9	0.918	0.942	0.921
QSVM + Graph OM (proposed)	Quantum ML + Ontology	95.1 ± 0.8	0.927	0.951	0.934

All methods above were evaluated on OWLS-TC v4 using identical experimental conditions.

## 5. Conclusion

This study explored the comparative performance of classical and quantum machine learning (QML) models for the task of Semantic Web Services (SWS) discovery, with a focus on evaluating how ontological matching techniques can enhance prediction accuracy. Rather than proposing new algorithms, the primary aim of this work was to analyze the behavior and effectiveness of existing QML models—such as Quantum k-Nearest Neighbors (QkNN), Quantum Decision Trees, Quantum Random Forest, and Quantum Support Vector Machines (QSVM)—in the context of semantic service discovery tasks. Our findings indicate that the integration of semantic ontological matching methods can significantly improve the performance of both classical and quantum classifiers. Among the models studied, Quantum Random Forest and QkNN demonstrated superior results, particularly when augmented with ontological reasoning mechanisms. QkNN, for instance, showed a measurable increase in classification accuracy after incorporating ontology alignment. These results suggest that while quantum machine learning is still in its developmental stage, it holds potential for boosting discovery tasks in structured and semantically rich environments. However, the contribution of this work is not in introducing new quantum algorithms or novel mathematical frameworks. Instead, the value lies in offering an empirical benchmark and early insight into how current QML tools perform when integrated with semantic reasoning techniques. Despite encouraging results, this study acknowledges several limitations. The experiments were conducted using the publicly available OWLS-TC v4 benchmark, which provides a standardized and reproducible evaluation environment. Future work should investigate the framework on additional real-world service registries to further validate generalizability. The scope of the study was also confined to a specific subset of QML and classical models. Moreover, the quality and completeness of the ontologies used may have influenced the results of the matching algorithms. These factors introduce constraints on the generalizability of our findings. Future work should address these limitations by evaluating the proposed framework on real-world SWS datasets and investigating more advanced QML architectures, including hybrid quantum-classical approaches. Improving the semantic quality and domain coverage of the ontologies used in matching will also be vital for scaling the approach. Furthermore, expanding the analysis to include other metrics such as model robustness, computational cost, and scalability under real-time service loads would provide a more comprehensive understanding. In conclusion, this research offers a comparative exploration into the integration of QML and ontology-driven reasoning, laying groundwork for future, more rigorous investigations into quantum-enhanced semantic service discovery.

## Supporting information

S1 Table Confusion Matrices for Quantum Random Forest (QRF) Under All Four Ontology Matching Conditions. (DOCX)

S2 Table Per-Class Precision, Recall, and F1-Score for QRF with Graph-Based Ontology Matching. (DOCX)

S3 Table Hyperparameter Configurations and Software Specifications. (DOCX)

S4 Table Raw Per-Instance Prediction Outputs. (DOCX)
