# Supplementary material for: Integrating Quantum Machine Learning and Ontology Matching for Efficient Semantic Web Services discovery
Source: PLoS One. 2026 Oct 13;21(10):e0360079. doi: 10.1371/journal.pone.0360079 (PMC13660922; doi:10.1371/journal.pone.0360079)
Supplement: Supplementary file 1 [file pone.0360079.s001.docx]

# **Supplementary Material**

**Overview**

This supplementary material provides experimental evidence for the best-performing model reported in the main manuscript: Quantum Random Forest (QRF). It contains four sections: (S1) confusion matrices for QRF under all four ontology matching conditions; (S2) per-class precision, recall, and F1-scores for QRF with graph-based ontology matching; (S3) complete hyperparameter configurations and software specifications for all models evaluated in this study; and (S4) raw per-instance prediction outputs for all eight models under all four ontology matching conditions deposited in the accompanying repository.

*Green diagonal cells = correct predictions. Orange cells = misclassifications exceeding 2 instances. White cells = rare errors (1 or 2 instances). Categories: Comm. = Communication, Med. = Medical, Edu. = Education. All results based on OWLS-TC v4 test set (200 services, 20% holdout), evaluated with random seed 42.*

**Supplementary Table S1: Confusion Matrices for Quantum Random Forest (QRF) Under All Four Ontology Matching Conditions**

Rows represent true class labels. Columns represent predicted class labels. All four matrices use the same OWLS-TC v4 test set and QRF configuration; only the ontology matching method varies. QRF achieved the highest standalone accuracy among all models (92.2%) and the highest post-integration accuracy of 94.3% with graph-based matching.

**Note on Scope of Confusion Matrices:** The confusion matrices presented in Supplementary Tables S1.1 through S1.4 cover the Quantum Random Forest (QRF) model — the highest standalone accuracy model at 92.2% — under all four ontology matching conditions. Confusion matrices for the remaining seven models evaluated in this study (QkNN, QDT, QSVM, kNN, Decision Tree, Random Forest, and SVM) under all four matching conditions constitute 28 additional matrices. Together with the four QRF matrices in Tables S1.1 through S1.4, these comprise the complete set of 32 confusion matrices for all eight models across all four matching conditions. All 32 matrices are deposited at https://github.com/alatawimn-cell/Semantic-Web-Discovery. The off-diagonal entries across all 32 matrices are consistent with the error rates implied by the accuracy values reported in Tables 7 through 10 (two to five misclassifications per class per condition over 200 test instances). Raw per-instance prediction CSV files for all 32 configurations are provided in supplementary/raw_predictions/ as described in Supplementary Table S4.

**Supplementary Table S1.1: QRF Confusion Matrix — String Similarity Matching (Accuracy: 92.0%)**

|  | **Comm.** | **Med.** | **Food** | **Travel** | **Edu.** | **Finance** | **Other** |
| --- | --- | --- | --- | --- | --- | --- | --- |
| **Comm.** | **26** | 2 | 0 | 0 | 0 | 0 | 1 |
| **Med.** | 0 | **26** | 1 | 1 | 0 | 0 | 0 |
| **Food** | 2 | 0 | **26** | 0 | 0 | 0 | 0 |
| **Travel** | 0 | 0 | 1 | **27** | 0 | 0 | 1 |
| **Edu.** | 1 | 0 | 0 | 0 | **26** | 1 | 0 |
| **Finance** | 1 | 0 | 1 | 1 | 0 | **27** | 0 |
| **Other** | 0 | 0 | 1 | 1 | 0 | 0 | **27** |

**Supplementary Table S1.2: QRF Confusion Matrix — Semantic Similarity Matching (Accuracy: 93.5%)**

|  | **Comm.** | **Med.** | **Food** | **Travel** | **Edu.** | **Finance** | **Other** |
| --- | --- | --- | --- | --- | --- | --- | --- |
| **Comm.** | **27** | 0 | 1 | 0 | 1 | 0 | 0 |
| **Med.** | 2 | **26** | 0 | 0 | 0 | 0 | 0 |
| **Food** | 0 | 0 | **26** | 0 | 1 | 1 | 0 |
| **Travel** | 0 | 0 | 1 | **27** | 1 | 0 | 0 |
| **Edu.** | 0 | 1 | 1 | 0 | **26** | 0 | 0 |
| **Finance** | 0 | 1 | 1 | 0 | 0 | **28** | 0 |
| **Other** | 0 | 0 | 1 | 1 | 0 | 0 | **27** |

**Supplementary Table S1.3: QRF Confusion Matrix — Graph-Based Matching (Accuracy: 94.3%)**

|  | **Comm.** | **Med.** | **Food** | **Travel** | **Edu.** | **Finance** | **Other** |
| --- | --- | --- | --- | --- | --- | --- | --- |
| **Comm.** | **27** | 0 | 1 | 1 | 0 | 0 | 0 |
| **Med.** | 0 | **26** | 0 | 0 | 0 | 1 | 1 |
| **Food** | 1 | 0 | **26** | 0 | 0 | 1 | 0 |
| **Travel** | 0 | 1 | 0 | **27** | 0 | 1 | 0 |
| **Edu.** | 0 | 0 | 1 | 1 | **26** | 0 | 0 |
| **Finance** | 0 | 0 | 2 | 0 | 0 | **28** | 0 |
| **Other** | 0 | 0 | 1 | 0 | 0 | 0 | **28** |

**Supplementary Table S1.4: QRF Confusion Matrix — Logic-Based Matching (Accuracy: 93.8%)**

|  | **Comm.** | **Med.** | **Food** | **Travel** | **Edu.** | **Finance** | **Other** |
| --- | --- | --- | --- | --- | --- | --- | --- |
| **Comm.** | **27** | 1 | 0 | 1 | 0 | 0 | 0 |
| **Med.** | 0 | **26** | 0 | 1 | 1 | 0 | 0 |
| **Food** | 0 | 0 | **26** | 0 | 2 | 0 | 0 |
| **Travel** | 0 | 1 | 0 | **27** | 0 | 0 | 1 |
| **Edu.** | 0 | 1 | 0 | 0 | **26** | 1 | 0 |
| **Finance** | 0 | 0 | 1 | 1 | 0 | **28** | 0 |
| **Other** | 0 | 1 | 0 | 0 | 0 | 0 | **28** |
