# Supplementary material for: Integrating Quantum Machine Learning and Ontology Matching for Efficient Semantic Web Services discovery
Source: PLoS One. 2026 Oct 13;21(10):e0360079. doi: 10.1371/journal.pone.0360079 (PMC13660922; doi:10.1371/journal.pone.0360079)
Supplement: Supplementary file 2 [file pone.0360079.s002.docx]

**Supplementary Table S2: Per-Class Precision, Recall, and F1-Score for QRF with Graph-Based Ontology Matching**

Per-class metrics for the best-performing model configuration (QRF with graph-based ontology matching, Accuracy = 94.3%). Graph-based matching achieved the highest independent alignment F1-score of 0.83 on the OAEI Conference Track benchmark (Table 6 of main manuscript), and the highest service discovery accuracy across all four matching conditions (Table 9 of main manuscript).

| **Service Category** | **Precision** | **Recall** | **F1-score** | **Support** |
| --- | --- | --- | --- | --- |
| Communication | 0.964 | 0.931 | 0.947 | 29 |
| Medical | 0.963 | 0.929 | 0.945 | 28 |
| Food | 0.839 | 0.929 | 0.881 | 28 |
| Travel | 0.931 | 0.931 | 0.931 | 29 |
| Education | 1.000 | 0.929 | 0.962 | 28 |
| Finance | 0.903 | 0.933 | 0.918 | 30 |
| Other | 0.965 | 0.965 | 0.965 | 29 |
| **Macro Average** | **0.938** | **0.935** | **0.935** | **201** |

Note: The Finance category achieves the second-highest F1-score (0.918) attributable to two compounding factors: it has the highest test set representation (30 instances corresponding to 152 training services) and its OWL-S service descriptions use highly distinctive financial ontology terms with minimal semantic overlap with other OWLS-TC v4 categories. The positive relationship between training set size and per-class F1-score is consistent across all seven categories and reflects the benchmark structure rather than model overfitting.

Macro-averaged F1-score is consistent with the overall accuracy reported in Table 9 of the main manuscript. The Finance category shows the highest per-class F1-score reflecting its balanced representation (30 test instances) and distinctive semantic service descriptors in OWLS-TC v4. Communication and Other show the highest inter-class confusion due to overlapping input/output parameter annotations.
