# Supplementary material for: Integrating Quantum Machine Learning and Ontology Matching for Efficient Semantic Web Services discovery
Source: PLoS One. 2026 Oct 13;21(10):e0360079. doi: 10.1371/journal.pone.0360079 (PMC13660922; doi:10.1371/journal.pone.0360079)
Supplement: Supplementary file 3 [file pone.0360079.s003.docx]

**Supplementary Table S3: Hyperparameter Configurations and Software Specifications**

All hyperparameter settings, software versions, and implementation details for every model evaluated in this study. All settings were held constant across all four ontology matching conditions to ensure fair comparison. The same configuration was used for both the classification results (Tables 7 through 10) and retrieval metric results (Table 19) reported in the main manuscript.

| **Model** | **Parameter** | **Value** | **Justification** |
| --- | --- | --- | --- |
| **All QML Models** | Feature encoding | Amplitude via ZZFeatureMap | *Amplitude encoding via ZZFeatureMap is the standard approach for continuous feature vectors in quantum kernel methods, following Havlicek et al. [27]* |
| All QML Models | PCA components (n) | 10 qubits | *PCA retains greater than 95% of cumulative explained variance in 128-dimensional TF-IDF vectors; n=10 satisfies 2^n = 1,024 amplitude encoding constraint* |
| All QML Models | ZZFeatureMap depth | 2 | *Depth 2 provides sufficient feature interaction expressivity while avoiding barren plateau optimization issues associated with deeper circuits, as established by Havlicek et al. [27]* |
| All QML Models | Entanglement | Full pairwise CNOT | *Full pairwise CNOT entanglement enables non-classical correlations between all feature dimensions, as justified by Schuld and Petruccione [26] for quantum kernel computation* |
| All QML Models | Measurement shots | 1,024 | *1,024 shots reduces kernel estimation variance below 0.01 for 10-qubit circuits based on standard shot noise analysis* |
| All QML Models | Backend | Qiskit Aer statevector | *Noiseless simulation* |
| All QML Models | Framework | PennyLane 0.35 + Qiskit 0.42 | *Hybrid quantum-classical workflow* |
| **QkNN** | k neighbors | 5 | *Standard default, validated via CV* |
| QkNN | Distance metric | Quantum cosine similarity | *From quantum kernel inner product* |
| **QDT** | Max depth | 10 | *Prevents overfitting on 800 samples* |
| QDT | Min samples split | 5 | *Reliable split statistics* |
| **QRF** | Number of trees (T) | 100 | *Standard ensemble size* |
| QRF | Max features | sqrt(d) | *Standard RF heuristic* |
| **QSVM** | Kernel | Quantum K(xi,xj)=\|<psi(xi)\|psi(xj)>\|^2 | *Quantum feature map replaces RBF* |
| QSVM | Regularization C | 1.0 | *Balanced margin vs. misclassification* |
| **Classical kNN** | k neighbors | 5 | *Same as QkNN for fair comparison* |
| **Classical DT** | Max depth | 10 | *Same as QDT for fair comparison* |
| **Classical RF** | Number of trees | 100 | *Same as QRF for fair comparison* |
| **Classical SVM** | Kernel | RBF | *Standard nonlinear baseline kernel* |
| **All Models** | CV folds | 5 | *5-fold cross-validation throughout* |
| All Models | Random seed | 42 | *Fixed for full reproducibility* |
| All Models | Train/Test split | 80/20 | *Standard on 1,000 OWLS-TC v4 services* |
| All Models | Scikit-learn version | 1.3.1 | *Classical model implementations* |
| All Models | Python version | 3.10 | *Compatible with PennyLane and Qiskit* |

All software dependencies are provided in a requirements.txt file in the accompanying repository with the following pinned versions: pennylane==0.35.0 (quantum simulation); qiskit==0.42.0 (IBM quantum backend); qiskit-aer (quantum simulator); scikit-learn==1.3.1 (classical ML and TF-IDF); numpy; pandas; matplotlib; seaborn; nltk (Wu-Palmer semantic similarity via WordNet); rdflib (RDF/OWL ontology parsing); sparqlwrapper (SPARQL query execution for logic-based matching); torch and torch-geometric (GraphSAGE baseline). A setup.py installation script and a config/config.yaml file centralizing all hyperparameters are also included: PCA components = 10; ZZFeatureMap depth = 2; entanglement = full pairwise CNOT; measurement shots = 1,024; circuit depths: QSVM = 6, QkNN = 6, QDT = 4, QRF = 4; k = 5; QRF trees = 100; CV folds = 5; random seed = 42; train/test split = 80/20; similarity thresholds: string (Jaccard) = 0.6, semantic (Wu-Palmer) = 0.5, graph (GED) = 0.4.
