# Supplementary material for: Integrating Quantum Machine Learning and Ontology Matching for Efficient Semantic Web Services discovery
Source: PLoS One. 2026 Oct 13;21(10):e0360079. doi: 10.1371/journal.pone.0360079 (PMC13660922; doi:10.1371/journal.pone.0360079)
Supplement: Supplementary file 4 [file pone.0360079.s004.docx]

**Supplementary Table S4: Raw Per-Instance Prediction Outputs**

Raw per-instance prediction outputs for all eight models under all four ontology matching conditions (32 experimental configurations) evaluated on the 200-instance OWLS-TC v4 test set (20% holdout, random seed 42) are deposited as CSV files in supplementary/raw_predictions/ in the accompanying repository at https://github.com/alatawimn-cell/Semantic-Web-Discovery. Each CSV file is named according to the convention ModelName_MatchingCondition_predictions.csv (for example, QSVM_graph_predictions.csv) and contains five columns: (1) service_id — the OWLS-TC v4 service identifier; (2) true_label — ground-truth functional category from OWLS-TC v4 annotations; (3) predicted_label — model output class; (4) confidence_score — model confidence or probability estimate for the predicted class; and (5) matching_method — the ontology matching condition applied (string, semantic, graph, or logic-based). These files enable full independent verification of all classification results reported in Tables 7 through 10 and all retrieval metric results reported in Table 19.

**Supplementary Table S4**: Raw Per-Instance Prediction Output Files

| **File Name** | **Model** | **Matching Condition** | **Instances** | **Columns** |
| --- | --- | --- | --- | --- |
| QSVM_string_predictions.csv | QSVM | String | 200 | service_id, true_label, predicted_label, confidence_score, matching_method |
| QSVM_semantic_predictions.csv | QSVM | Semantic | 200 | service_id, true_label, predicted_label, confidence_score, matching_method |
| QSVM_graph_predictions.csv | QSVM | Graph | 200 | service_id, true_label, predicted_label, confidence_score, matching_method |
| QSVM_logic_predictions.csv | QSVM | Logic | 200 | service_id, true_label, predicted_label, confidence_score, matching_method |
| QRF_string_predictions.csv | QRF | String | 200 | service_id, true_label, predicted_label, confidence_score, matching_method |
| QRF_semantic_predictions.csv | QRF | Semantic | 200 | service_id, true_label, predicted_label, confidence_score, matching_method |
| QRF_graph_predictions.csv | QRF | Graph | 200 | service_id, true_label, predicted_label, confidence_score, matching_method |
| QRF_logic_predictions.csv | QRF | Logic | 200 | service_id, true_label, predicted_label, confidence_score, matching_method |
| QkNN_string_predictions.csv | QkNN | String | 200 | service_id, true_label, predicted_label, confidence_score, matching_method |
| QkNN_semantic_predictions.csv | QkNN | Semantic | 200 | service_id, true_label, predicted_label, confidence_score, matching_method |
| QkNN_graph_predictions.csv | QkNN | Graph | 200 | service_id, true_label, predicted_label, confidence_score, matching_method |
| QkNN_logic_predictions.csv | QkNN | Logic | 200 | service_id, true_label, predicted_label, confidence_score, matching_method |
| QDT_string_predictions.csv | QDT | String | 200 | service_id, true_label, predicted_label, confidence_score, matching_method |
| QDT_semantic_predictions.csv | QDT | Semantic | 200 | service_id, true_label, predicted_label, confidence_score, matching_method |
| QDT_graph_predictions.csv | QDT | Graph | 200 | service_id, true_label, predicted_label, confidence_score, matching_method |
| QDT_logic_predictions.csv | QDT | Logic | 200 | service_id, true_label, predicted_label, confidence_score, matching_method |
| kNN_string_predictions.csv | kNN | String | 200 | service_id, true_label, predicted_label, confidence_score, matching_method |
| kNN_semantic_predictions.csv | kNN | Semantic | 200 | service_id, true_label, predicted_label, confidence_score, matching_method |
| kNN_graph_predictions.csv | kNN | Graph | 200 | service_id, true_label, predicted_label, confidence_score, matching_method |
| kNN_logic_predictions.csv | kNN | Logic | 200 | service_id, true_label, predicted_label, confidence_score, matching_method |
| DT_string_predictions.csv | Decision Tree | String | 200 | service_id, true_label, predicted_label, confidence_score, matching_method |
| DT_semantic_predictions.csv | Decision Tree | Semantic | 200 | service_id, true_label, predicted_label, confidence_score, matching_method |
| DT_graph_predictions.csv | Decision Tree | Graph | 200 | service_id, true_label, predicted_label, confidence_score, matching_method |
| DT_logic_predictions.csv | Decision Tree | Logic | 200 | service_id, true_label, predicted_label, confidence_score, matching_method |
| RF_string_predictions.csv | Random Forest | String | 200 | service_id, true_label, predicted_label, confidence_score, matching_method |
| RF_semantic_predictions.csv | Random Forest | Semantic | 200 | service_id, true_label, predicted_label, confidence_score, matching_method |
| RF_graph_predictions.csv | Random Forest | Graph | 200 | service_id, true_label, predicted_label, confidence_score, matching_method |
| RF_logic_predictions.csv | Random Forest | Logic | 200 | service_id, true_label, predicted_label, confidence_score, matching_method |
| SVM_string_predictions.csv | SVM | String | 200 | service_id, true_label, predicted_label, confidence_score, matching_method |
| SVM_semantic_predictions.csv | SVM | Semantic | 200 | service_id, true_label, predicted_label, confidence_score, matching_method |
| SVM_graph_predictions.csv | SVM | Graph | 200 | service_id, true_label, predicted_label, confidence_score, matching_method |
| SVM_logic_predictions.csv | SVM | Logic | 200 | service_id, true_label, predicted_label, confidence_score, matching_method |
